# ACE2/Ang-(1-7)/Mas1 axis and the vascular system: vasoprotection to COVID-19-associated vascular disease

**DOI:** 10.1042/CS20200480

**Published:** 2021-01-29

**Authors:** Jithin Kuriakose, Augusto C. Montezano, Rhian M. Touyz

**Affiliations:** Institute of Cardiovascular and Medical Sciences, University of Glasgow, Glasgow, Scotland, United Kingdom

**Keywords:** angiotensin converting enzyme 2, COVID-19, endothelial cells, vascular smooth muscle

## Abstract

The two axes of the renin–angiotensin system include the classical ACE/Ang II/AT1 axis and the counter-regulatory ACE2/Ang-(1-7)/Mas1 axis. ACE2 is a multifunctional monocarboxypeptidase responsible for generating Ang-(1-7) from Ang II. ACE2 is important in the vascular system where it is found in arterial and venous endothelial cells and arterial smooth muscle cells in many vascular beds. Among the best characterized functions of ACE2 is its role in regulating vascular tone. ACE2 through its effector peptide Ang-(1-7) and receptor Mas1 induces vasodilation and attenuates Ang II-induced vasoconstriction. In endothelial cells activation of the ACE2/Ang-(1-7)/Mas1 axis increases production of the vasodilator’s nitric oxide and prostacyclin’s and in vascular smooth muscle cells it inhibits pro-contractile and pro-inflammatory signaling. Endothelial ACE2 is cleaved by proteases, shed into the circulation and measured as soluble ACE2. Plasma ACE2 activity is increased in cardiovascular disease and may have prognostic significance in disease severity. In addition to its enzymatic function, ACE2 is the receptor for severe acute respiratory syndrome (SARS)-coronavirus (CoV) and SARS-Cov-2, which cause SARS and coronavirus disease-19 (COVID-19) respectively. ACE-2 is thus a double-edged sword: it promotes cardiovascular health while also facilitating the devastations caused by coronaviruses. COVID-19 is associated with cardiovascular disease as a risk factor and as a complication. Mechanisms linking COVID-19 and cardiovascular disease are unclear, but vascular ACE2 may be important. This review focuses on the vascular biology and (patho)physiology of ACE2 in cardiovascular health and disease and briefly discusses the role of vascular ACE2 as a potential mediator of vascular injury in COVID-19.

## Introduction

**Figure 1:**
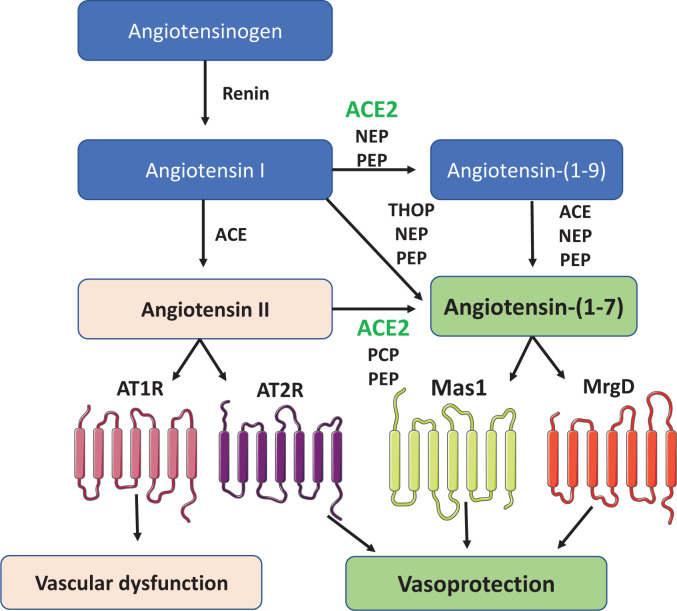
Mechanisms involved in the generation and catabolism of angiotensin peptides, including Ang-(1-7) Renin converts angiotensinogen to angiotensin I, which is cleaved by ACE to generate angiotensin II. ACE2, PCP and PEP catalyse the conversion of angiotensin II to angiotensin-(1-7). Angiotensin-(1-7) can also be formed from angiotensin I by ACE2, which involves the production of the intermediate form angiotensin-(1-9) and its cleavage by ACE, NEP or PEP. Angiotensin II signals through AT1 and AT2 receptor, while angiotensin-(1-7) signals through Mas1 and MrgD. ACE, angiotensin-converting enzyme; ACE2, angiotensin-converting enzyme 2; AT1R, angiotensin II type 1 receptor; AT2R, angiotensin II type 2 receptor; NEP, neutral endopeptidase; Mas1, Mas1 receptor; MrgD, Mas-related-G-protein-coupled receptor D; PCP, prolyl carboxypeptidase; PEP, prolyl oligopeptidase; THOP, thimet oligopeptidase.

The renin–angiotensin system (RAS) plays a pivotal role in the regulation of blood pressure, electrolyte and water homeostasis, vascular tone and cardiovascular and renal health [1]. It is a peptidergic system comprising a circulating and a tissue component [2]. Activation is initiated with the release of liver-derived angiotensinogen into the circulation, where it is cleaved by kidney-derived renin into angiotensin I (Ang I) [3], which undergoes further cleavage to angiotensin II (Ang II) by angiotensin-converting enzyme (ACE), expressed mainly in pulmonary endothelial cells (Figure 1). In addition to the classical Ang I/ACE/Ang II pathway, an alternate pathway was identified when ACE2, a homologue of ACE, was discovered [4,5]. ACE2 hydrolyses multiple peptides, including Ang I and Ang II to produce Ang-(1-9) and Ang-(1-7) respectively [6].

Ang II is the main effector peptide of the RAS and signals via Ang II type 1 (AT1R) and Ang II type 2 receptors (AT2R) [7]. Ang II binding to the AT1R leads to coupling of G proteins (G_i_/G_o_, G_12_/G_13_ and G_q_/G_11_), second messenger signaling through Ca^2+^ and reactive oxygen species (ROS) generation with subsequent activation of downstream signaling molecules including myosin light chain kinase (MLCK), calcium channels, phospholipases, Rho kinase, mitogen-activated protein kinases (MAPK), serine threonine kinases (PI3K, Akt, PKC) and tyrosine kinases (c-Src, JAK/STAT, epidermal growth factor receptor (EGFR)) [8]. Ang II binding to the AT2R induces endothelial nitric oxide synthase (eNOS) activation, nitric oxide (NO) generation, protein tyrosine phosphatase activation and sphingolipid signaling leading to vasorelaxation, decreased vascular tone and natriuresis [9]. The major (patho)physiological actions of Ang II, including vasoconstriction, inflammation and fibrosis, are mediated via AT1Rs [10,11]. In general, AT2R-mediated actions oppose those of the AT1R; however, the exact physiological role of AT2Rs in humans still remains unclear.

Whereas ACE cleaves the decapeptide Ang I to the octapeptide Ang II, ACE2 cleaves Ang I and Ang II to form Ang-(1-9) and Ang-(1-7), respectively [4,12]. ACE2 is a negative regulator of the RAS because it decreases Ang II levels and increases Ang-(1-7) and Ang-(1-9), which are vasodepressor peptides that counteract the vasoconstrictor actions of Ang II [13,1]. Beyond Ang I and Ang II, ACE2 has numerous biological substrates including (des-Arg9)-bradykinin, an agonist of the B1 receptor, apelin-13, a vasoconstrictor and other non-RAS peptides including kinetensin, dynorphin A and neurotensin [14]. Ang-(1-7) is also generated from Ang I by thimet oligopeptidase (THOP1), prolyl oligopeptidase (PEP) and neutral endopeptidase (NEP), while carboxypeptidase A and prolyl carboxypeptidase (PCP) generate Ang-(1-7) from Ang II [15–17]. Ang-(1-7) signals mainly through receptor Mas1 and to a lesser extent through Mas related G protein-coupled receptor D (MrgD) [18,19]. Activation of the ACE2/Ang-(1-7)/Mas1 pathway has vasodilatory, anti-proliferative, anti-inflammatory and anti-fibrotic effects and accordingly has been described as the counter-regulatory arm of the RAS, especially in the vascular system where it protects against increased vascular tone and pathological remodelling and inflammation [20]. Accordingly, there is increasing interest in targeting ACE2/Ang-(1-7)/Mas1 as a vasoprotective strategy in cardiovascular disease. In addition to its function as a master negative regulator of the RAS, ACE2 acts as the functional receptor for severe acute respiratory syndrome (SARS) coronaviruses (SARS-CoV) including SARS-CoV-2, the cause of coronavirus disease-19 (COVID-19) [21–23].

While ACE2 is widely expressed and has multiple functions influencing various (patho)physiological systems, this review focuses primarily on ACE2 in the vascular system and discusses the role of Ang-(1-7) as the primary peptide effector of ACE2. In addition, we briefly discuss the putative role of vascular ACE2 in the cardiovascular sequelae of COVID-19. It should be highlighted that the field of vascular ACE2, SARS-CoV-2 and COVID-19 is still immature, with many gaps in knowledge. Accordingly, here we only provide some introductory comments on vascular ACE2 and COVID-19. Further original research is needed for a comprehensive review on the topic.

## ACE2-a primer

ACE2 shares ∼42% homology with ACE and similar to ACE is a transmembrane protein with the extracellular domain comprising catalytic function. In humans, ACE2 protein is encoded by the *Ace2* gene on the X-chromosome and is located near a quantitative trait locus linked to hypertension [24]. Unlike ACE, ACE2 does not cleave bradykinin, and it is not inhibited by classical ACE inhibitors [4,25]. The membrane-bound ectodomain of ACE2 is cleaved by tumour necrosis factor-alpha-converting enzyme (TACE/ADAM17), resulting in shedding of ACE2 into the circulation [26]. This is the soluble form of ACE2 and can be measured in plasma. The relative amount of soluble ACE2 to membrane-bound ACE2 is very low, and the physiological role of soluble ACE2 still remains unclear, although it may play a role in the degradation of circulating, but not tissue, Ang II to Ang-(1-7) [27]. Plasma ACE2 activity is low in healthy individuals, possibly due to an endogenous inhibitor [28,29]. However, in cardiovascular disease shedding of ACE2 is increased and plasma ACE2 levels and activity may be increased [30,31]. Plasma ACE2 levels are higher in men than women in health and disease [32] and could relate to the fact that *Ace2* is an X-linked gene that might reflect sex-specific differences in control of ACE2 expression and activity.

ACE2 is a multifunctional protein. Beyond its catalytic function generating the peptides Ang-(1-9) and Ang-(1-7), it has noncatalytic actions including the regulation of renal amino acid transport, control of intestinal neutral amino acid transport, pancreatic insulin secretion and coronavirus receptor [33]. As the functional receptor of SARS-CoV and SARS-CoV-2, ACE2 plays a critical role in the pathophysiology of SARS and COVID-19 [20–22]. ACE2 may also be a signaling molecule involved in outside-in signalling [34,35]. The cytosolic domain possesses numerous phosphorylation sites and interacts with calmodulin, which may inhibit shedding of the ectodomain [34,35]. ACE2 has a widespread distribution, and its biological functions likely relate to its expression pattern and activation state in different tissues and organs.

## Expression of ACE2 in the vascular system

ACE2 is ubiquitously expressed in human tissues in normal and pathological conditions. In the vascular system, ACE2 is localized in arterial and venous endothelial cells and arterial smooth muscle cells in many organs (Table 1) [12,36–45]. ACE2 protein has also been detected in human atherosclerotic arteries and vascular macrophages [36]. In patients with ischemic heart disease undergoing coronary artery bypass surgery, immunohistochemical analysis showed abundant ACE2 positive cells in diseased vessels, particularly in the neo-intima and media, and in newly formed angiogenic vessels and vasa vasorum, indicating a potential role for ACE2 in pathological conditions [38]. In the human heart, single nuclei RNA sequencing demonstrated cell type-specific expression of ACE2, which was identified in cardiomyocytes, fibroblasts, endothelial cells, smooth muscle cells and pericytes [46]. In patients with heart disease, expression of ACE2 was increased in cardiomyocytes and endothelial cells, whereas fibroblast ACE2 expression was reduced compared with healthy controls [46]. Cardiomyocyte ACE2 content is also increased in patients with aortic stenosis and heart failure [47].

**Table 1:** Table listing the studies which have reported the expression of ACE2 and Mas1 in endothelial cells of different vascular beds

Vascular bed	ACE2/Mas1 expression (Protein/mRNA/Activity)	Species	References
Carotid artery	ACE2 mRNA	Human	[36]
Colon and Brain	ACE2 protein	Human	[37]
Liver and Spleen	ACE2 protein	Human	[37]
Oral mucosa and small intestine	ACE2 protein	Human	[37]
Heart, kidney and Testis	ACE2 protein	Human	[12]
Heart	ACE2 mRNA, protein and activity	Rat	[38]
Heart	ACE2 protein and mRNA	Human	[38]
Pancreas	ACE2 protein	Human	[39]
Thyroid and parathyroid gland	ACE2 protein	Human	[39]
Adrenal gland and fallopian tube	ACE2 protein	Human	[39]
Umbilical cord	ACE2 protein and mRNA	Human	[40]
Lungs	ACE2 protein	Rat	[41]
Vasa Vasorum	ACE2 protein	Human	[42]
Eye	Mas1 protein and mRNA	Mouse	[43]
Lungs	Mas1 mRNA and protein	Human	[44]
Brain	Mas1 mRNA	Rat	[45]

Abbreviations: ACE-2, angiotensin-converting enzyme 2; Mas1, Mas1 receptor; mRNA, messenger RNA

Disruption of ACE/ACE2 balance in the cardiovascular system has been implicated in hypertension [28]. However, ACE2 as a potential therapeutic target in hypertension has been disappointing because ACE2 inhibitors do not increase blood pressure significantly in experimental models and agents that enhance ACE2 activity, including recombinant human ACE2 (rhACE2), have failed to consistently reduce blood pressure in hypertensive models and humans [28,48].

## Regulation of vascular ACE2

Mechanisms that control ACE2 in the cardiovascular system are complex and multifactorial. ACE is regulated at the transcriptional and post-transcriptional levels. Molecular elements such as miRNAs (miR-421) [49], transcription factors (C/EBPβ) [50] and signalling molecules (sirtuin-1) [51] have been shown to influence ACE2 expression. ACE2 is also regulated by Ang II through AT1 and AT2 receptors. Increased Ang II/AT1R signaling through ERK1/2 and p38MAPK in cardiovascular and kidney tissue down-regulates ACE2 while expression of ACE is increased [52]. On the other hand, activation of AT2R increases expression and activity of ACE2 in endothelial cells [53].

Aldosterone, through mineralocorticoid receptor activation, may also regulate ACE2 [54]. Aldosterone does not seem to influence ACE2 enzymatic activity but influences its expression. In neonatal cardiomyocytes, aldosterone, but not Ang II, decreased mRNA expression of ACE2, with an associated increase in ACE expression [55]. This imbalance between ACE2 and ACE may contribute to pathological cardiovascular remodelling. These *in vitro* findings were recapitulated in *in vivo* studies where aldosterone infusion in rats induced a significant decrease in renal ACE2 expression, particularly in the brush border membrane of the proximal convoluted tubules [56]. This was mediated through mineralocorticoid receptor signalling via proinflammatory NFkB pathways. Spironolactone treatment normalised ACE2 expression in aldosterone-treated rats, supporting the role of mineralocorticoid receptors in aldosterone-mediated ACE2 regulation [56].

Besides humoral mediators, vascular ACE2 is regulated by mechanical factors such as shear stress and stretch. In vascular smooth muscle cells, stretch increased the promoter activity of ACE2 but did not affect its mRNA stability, indicating that mechanical stretch influences ACE2 primarily at the transcriptional level [57]. Molecular processes underlying this involve activator protein-1 (AP-1), which directly interacts with ACE2. NF-κB seems to be a negative regulator of ACE2 in an indirect manner, since the ACE2 promoter site lacks a binding site for NF-κB. PKCβII and JNK1/2 have also been implicated in stretch-mediated ACE2 expression. It has been proposed that physiological stretch up-regulates vascular smooth muscle cell ACE2, which inhibits proliferation and migration, likely through increased Ang-(1-7)/Mas1 signalling [57].

## Vascular ACE2 regulation and inhibitors of the RAS

Based on the premise that inhibition of the ACE/Ang II/AT1R pathway potentiates ACE2 actions and activation of the counter-regulatory system, it has been suggested that drugs that inhibit the RAS will influence ACE2 expression and activity [58–60]. Clinical studies demonstrated increased expression of ACE2 in cardiomyocytes and vascular cells in hearts from patients who were treated with ACE inhibitors (ACEi) compared with those treated with angiotensin receptor blockers (ARB) [59]. Moreover, the ACE:ACE2 ratio was four-fold higher in ACEi-treated patients versus healthy individuals and may represent an imbalance between the injurious and protective arms of the RAS. In ACEi-treated patients with coronary artery disease, Ang-(1-7) production was not altered, indicating lack of ACE2 modulation [59,60]. Similarly, in cross-sectional studies involving patients with heart failure, aortic stenosis, coronary artery disease and atrial fibrillation, the use of ACEi or ARBs did not affect plasma ACE2 activity in comparison with untreated patients [61–63]. In contrast, other studies in patients with hypertension showed that olmesartan, but not losartan, candesartan, valsartan or telmisartan, increased urinary ACE2, indicating a possible drug-specific effect [64].

In rodent models of cardiovascular injury, ACEi and ARBs have been associated with both up-regulation and down-regulation of circulating and cardiovascular ACE2 [65–69]. Early studies in rodents showed that ACEi and ARBs enhance tissue ACE2 expression and increase plasma Ang-(1-7) levels. Studies in experimental models of hypertension, coronary artery ligation, atherosclerosis and diabetes, reported that ACEi and ARB treatment increases ACE2 gene expression, plasma ACE2 levels and ACE2 activity [70]. Based on these findings, it was suggested that beneficial clinical effects of ACEi and ARBs may relate, at least in part, to inhibition of the ACE/Ang II pathway and activation of the ACE2/Ang-(1-7) pathway [69,70]. Supporting this, experimental studies showed that the Mas1 receptor antagonist (A779) blocked ACE2/Ang-(1-7) responses of ACEi and ARBs [71]. While there is strong evidence that inhibitors of the RAS increase ACE2 expression at the gene level in vessels, heart and kidneys, this has not been consistently demonstrated at the protein level. The functional significance of this is unclear and reflects the complexity of the ACE2 system.

In addition to ACEis and ARBs, mineralocorticoid receptor antagonists have been shown to influence ACE2 expression. Spironolactone and eplerenone increase levels of ACE2 in plasma samples from patients with heart failure as well as in heart tissue from experimental models of cardiovascular disease [72]. These findings suggest that beneficial effects of mineralocorticoid receptor antagonists may be mediated, at least in part, by up-regulating ACE2 and the vasoprotective arm of the RAS.

## Functions of vascular ACE2

**Figure 2:**
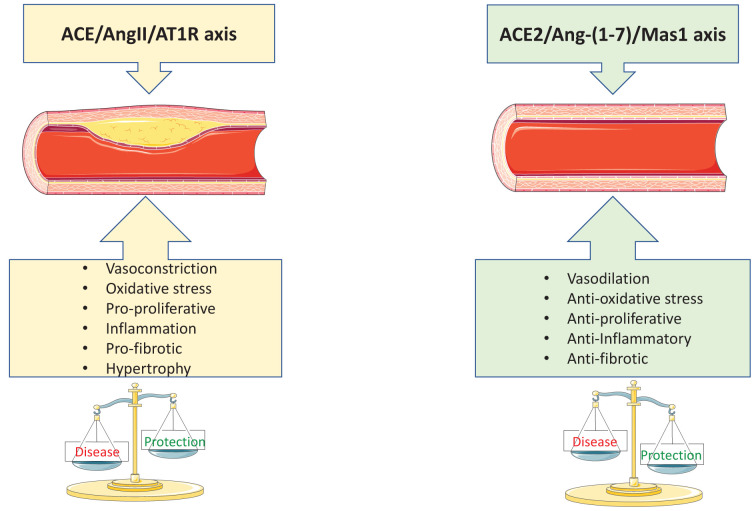
Schematic depicting the opposing effects of ACE/AngII/AT1R axis and ACE2/Ang-(1-7)/Mas1 axis in the vasculature Increased signalling through the ACE/AngII/AT1R axis promotes vascular injury while signalling through the ACE2/Ang-(1-7)/Mas1 axis leads to vasoprotection from the injury caused by ACE/AngII/AT1R axis stimulation. ACE, angiotensin-converting enzyme; ACE2, angiotensin converting enzyme two; Ang II, angiotensin two; Ang-(1-7), angiotensin 1 to 7; AT1R, angiotensin two type one receptor; Mas1, mas1 receptor.

The exact physiological function of vascular ACE2 is still unclear, but it may be an important regulator of normal endothelial and vascular smooth muscle cell function by fine-tuning and modulating vasoinjurious effects of Ang II (Figure 2). In pathological conditions, vascular ACE2 may play a role in preventing pathological vascular remodelling. In cultured human endothelial cells, activation of endogenous ACE2 inhibits Ang II-induced phosphorylation of ERK1/2 and c-Src, reduces Nox-derived ROS generation and increases phosphorylation of eNOS and production of NO, processes that promote vasorelaxation [73,74]. Deletion of vascular smooth muscle cell ACE2 leads to phenotypic switching to a proliferative and pro-inflammatory state [75], processes that are reversed or prevented in cells in which ACE2 is up-regulated [76]. In human vascular smooth muscle cells, ACE2-derived Ang-(1-7) attenuates Ang II-mediated proliferation and inhibits vascular calcification and atherosclerosis [76,77].

ACE2 has also been shown to influence the function of endothelial progenitor cell-derived exosomes, especially related to phenotypic de-differentiation of vascular smooth muscle cells. In co-culture experiments with vascular smooth muscle cells, exosomes released from ACE2-expressing endothelial progenitor cells reduced proliferation, migration and pro-inflammatory effects of Ang II [78]. These ACE2-dependent processes attenuated Ang II-stimulated synthetic phenotypic switching through molecular events that downregulate NF-kB, thereby maintaining vascular smooth muscle cell function in its normal state [78].

## ACE2 and vascular pathophysiology in experimental models

Transgenic models have revealed the importance of vascular ACE2 in (patho)physiological conditions. One of the most important studies interrogating the specific role of vascular ACE2 in intact systems was that of Rentzsch and colleagues [79], where human ACE2 was expressed in a vascular smooth muscle cell-specific manner in spontaneously hypertensive stroke prone rats (SHRSP). In these transgenic hypertensive rats, amplification of vascular smooth muscle cell ACE2 was associated with significantly reduced blood pressure, decreased vasoconstrictor responses to Ang II and normalization of endothelium-dependent vasorelaxation, effects that were blocked by treatment with an ACE2 inhibitor [80]. Similar observations were made in lentiviral overexpression of ACE2 in spontaneously hypertensive rats (SHR) [80]. In SHR treated with the ACE2 activator xanthenone, development of hypertension was attenuated, and vascular remodelling was prevented [81]. SHR and Ang II-infused rats treated with rhACE2 had reduced blood pressure, decreased cardiovascular and renal oxidative stress, and suppressed pathological cardiac remodelling [82]. Together these findings suggest that in hypertension increased vascular ACE2 activity reduces blood pressure likely through a combination of increased degradation of Ang II and augmented Ang-(1-7) production, which improve vascular function. In Ang II-induced hypertension in mice, pretreatment with rhACE2 decreased blood pressure through processes that enhanced Ang II degradation rather than by increasing Ang-(1-7) levels [27]. Supporting these findings, genetic ablation of systemic ACE2 in C57BL/6 mice resulted in reduced NO bioavailability, increased oxidative stress and vascular dysfunction leading to significant elevation in blood pressure [83].

Vascular ACE2 may be particularly important in cardiovascular disorders associated with inflammation, fibrosis and remodelling. In atherosclerosis-prone ApoE knockout mice, genetic deletion of ACE2 accelerates vascular inflammation and plaque accumulation [84]. In rabbit aortic segments, ACE2 overexpression inhibited development of early atherosclerotic lesions by suppressing the proliferation and migration of vascular smooth muscle cells and by improving endothelial function [85. These studies indicated that the anti-atherosclerotic actions of ACE2 may result from reduced Ang II levels, increased Ang-(1-7) production, decreased ACE activity and down-regulation of AT1R signalling [84,85].

A vasoprotective role of ACE2 has also been demonstrated in pulmonary hypertension. In monocrotaline (MCT)-induced pulmonary hypertension in rats, oral administration of ACE2 bioencapsulated in plant cells attenuated the development and progression of pulmonary hypertension and prevented pulmonary vascular remodelling [86]. Chronic treatment with a synthetic ACE2 activator 1-[2-(dimetilamino)etil]amino]-4-(hidroximetil)-7-[(4-metilfenil)sulfonil]oxi]-9H-xantona-9 (XNT) attenuated MCT-induced pulmonary hypertension in rats with associated reduction in vascular wall thickness [87]. Pharmacological blockade of Mas1 receptor eliminated the cardioprotective effects of XNT, indicating that XNT elicits its protective effects primarily through the Ang-(1-7)/Mas1 pathway.

## ACE2 and vascular disease in humans

ACE2 mRNA and protein have been demonstrated in human atherosclerotic vessels, aortic aneurysms and coronary arteries in patients with heart failure [88–90]. ACE2 is expressed in both early and advanced atherosclerotic lesions [36]. While the content of ACE2 does not change during atherogenesis, ACE2 activity is lower in advanced plaques versus early atherosclerotic lesions. These findings suggest that ACE2 regulation changes during atherogenesis and that the vasoprotective function of ACE2 may be blunted with disease progression.

Vascular ACE2 has been identified in pathological vascular remodelling. In patients with bicuspid aortic valve, the ascending aorta is dilated, and this is associated with increased expression and activity of ACE2, findings that were recapitulated in murine models [88]. In this context, ACE2 up-regulation was considered as a compensatory mechanism. In human abdominal aortic aneurysms, ACE2 was identified in the intima, media and adventitial inflammatory cells [89]. Supporting these human findings, ACE2 deficiency in murine models promoted Ang II-induced development of aneurysms, effects that were prevented when ACE2 activity was increased pharmacologically with diminazine aceturate (DIZE) [89]. Hence therapeutic stimulation of ACE2 may protect against vascular remodelling in aortic disease and may have benefit in patients with aortic aneurysms.

## Endothelial ACE2 shedding, plasma soluble ACE2 and clinical implications

Shedding of ACE2 from endothelial cells by proteases releases the catalytically active ectodomain into the blood as soluble ACE2, which can be measured in plasma [28,29]. Clinical studies have shown that plasma ACE2 activity is increased in patients with hypertension, diabetes mellitus and chronic kidney disease [28]. However, some clinical studies failed to show increased plasma ACE2 activity in hypertension, while others showed decreased soluble ACE2 activity [28,91]. In pulmonary arterial hypertension plasma ACE2 activity was shown to be reduced [91]. This prompted a clinical study to assess whether human ACE2 therapy would improve pulmonary arterial hypertension. A single infusion of rhACE2 GSK2586881 resulted in significant improvement in cardiac output and pulmonary vascular resistance, suggesting that ACE2 upregulation may be a potential therapeutic strategy [91,92].

ACE2 shedding also occurs in healthy states. Isolated human CD34+ cells from healthy individuals, when exposed to hypoxic conditions, resulted in ACE2 ectodomain shedding [93]. This was further confirmed when blood flow restriction stimulated mobilization of hematopoietic stem/progenitor cells and increased circulating ACE2 levels in healthy subjects [94]. Thus ACE2 shedding may represent a physiological response to stress, which may become dysregulated in pathological conditions.

The functional role of soluble ACE2 is still unclear but may be a biomarker of underlying vascular stress or cardiovascular disease [47]. A recent association study examining the relationship between soluble ACE2 and plasma proteins revealed that plasma ACE2 associates with signaling proteins linked to clathrin-mediated endocytosis, actin cytoskeleton, eukaryotic initiation factor 2 (EIF2), protein ubiquitination and viral exit from host cells [90]. These proteins are involved in cellular endocytosis, exocytosis and intracellular trafficking of signaling molecules and may reflect cell damage and dysfunction in cardiovascular disease [90].

## Ang-(1-7)/Mas1 and vascular function

Ang-(1-7) is the major downstream peptide generated from Ang II by ACE2 catalytic activity. The majority of ACE2 vascular effects are mediated by Ang-(1-7) [95]. Ang-(1-7) functions in opposition to Ang II and in the vasculature promotes dilation [1]. Ang-(1-7) is anti-proliferative, anti-fibrotic, anti-inflammatory and anti-angiogenic, thereby maintaining vascular integrity and promoting vascular health [95,96]. These effects are mediated via the G protein coupled receptor Mas1, which signals through multiple pathways, including activation of Akt, increased NO generation, decreased ROS generation, and inhibition of Ang II-stimulated signalling pathways [1,6,73,74,95].

### Vasodilation

Vasodilation is the best characterized vascular action of Ang-(1-7) and is evident in large conduit vessels, resistance arteries and microvessels [96,97]. Ang-(1-7) synergistically increases vasodilation induced by bradykinin and ghrelin, and attenuates agonist-induced vasoconstriction [1,98]. While experimental studies clearly support a vasodilatory action of Ang-(1-7), this is less evident in human vessels. Some studies failed to show vasodilation in response to Ang-(1-7) in the forearm circulation of normotensive subjects and patients with hypertension [99,100], while others reported a dose-dependent augmentation of bradykinin vasodilation by Ang-(1-7) in forearm resistance vessels in healthy men, confirming the bradykinin-potentiating effect of Ang-(1-7) identified in experimental models [101]. In the forearm of normotensive patients as well as in mammary arteries *in vitro* Ang-(1-7) attenuates the vasoconstrictor effect of Ang II, but not noradrenaline [102,103].

### Antiproliferative and antifibrotic effects

In vascular smooth muscle cells, Ang-(1-7) opposes the mitogenic actions of Ang II by inhibiting activation of growth signalling pathways such as MAP kinases [104,105]. Ang-(1-7)/Mas1 also inhibits transactivation of growth factor receptors, such as EGFR, resulting in decreased activation of downstream signalling pathways involved in vascular smooth muscle cell proliferation and vascular hypertrophy [106]. In response to vascular injury, Ang-(1-7) reduces neointimal formation and pathological vascular remodelling [107]. In atherosclerotic prone ApoE knockout mice, Ang-(1-7) and AT1R blockade with losartan improved endothelial function, attenuated macrophage infiltration and inhibited vascular smooth muscle cell proliferation and migration [108]. In addition, Ang-(1-7) prevents vascular calcification and aneurysm formation by inhibiting osteogenic transition of vascular smooth muscle cells [109].

### Haematopoietic stem/progenitor cells and vascular repair

In addition to directly influencing vascular function, the ACE2/Ang-(1-7)/Mas1 system activates haematopoietic stem/progenitor cells, important in vascular repair [110–114]. ACE2/Ang-(1-7)/Mas1 activation promotes vasoreparative properties of vascular progenitor cells and reverses reparative dysfunction in pathological conditions [113–118]. In a mouse model of Type 2 diabetes, Ang-(1-7) treatment increased bone marrow and circulating levels of endothelial and mesenchymal stem cells [114]. Similar favourable responses were observed in a rat model of myocardial infarction treated with Ang-(1-7) and in a mouse model of ischaemic stroke where transfusion of lentivirus-ACE2-primed endothelial progenitor cells increased angiogenesis and cerebral microvascular density [115,116]. *In vitro* studies showed that activation of the ACE2/Ang-(1-7)/Mas1 axis stimulates vascular repair functions of CD34+ cells and enhances the reparative function of dysfunctional endothelial progenitor cells in diabetes [117].

## Ang-(1-7) signalling in vascular cells

### Endothelial cells, NO and prostacyclin production

**Figure 3:**
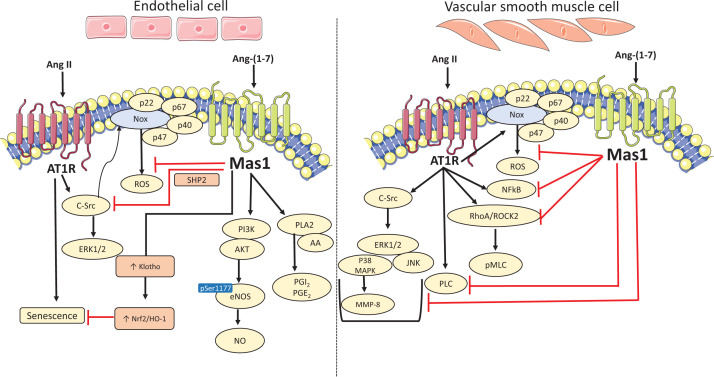
Vascular signalling of Ang-(1-7)/Mas1 in endothelial cells and vascular smooth muscle cells In endothelial cells, Ang-(1-7) activates eNOS via AKT to produce nitric oxide. Ang-(1-7) also stimulates production of vasodilators such as prostacyclin (PGI2) and prostaglandin (PGE2) via enhancement of phospholipase A2 (PLA2) activity and arachidonic acid (AA) release. Ang-(1-7) via Mas1 counter-regulates Ang II effects by increasing expression of anti-ageing protein klotho which consequently activates Nrf2/HO-1 pathway and activation of c-Src, ERK1/2 and NADPH oxidase (Nox) by enhancing SHP-2 phosphorylation. In smooth muscle cells, Ang-(1-7) via Mas1 receptor opposes Ang II-induced contraction and oxidative stress by attenuating RhoA/ROCK2 signalling, PLC and NADPH activation. Ang-(1-7) also suppresses Ang II-induced proliferation and inflammation by inhibiting MAPKs (ERK1/2, p38MAPK and JNK), MMP-8 and NFκB signalling. AKT, serine threonine specific protein kinase; Ang II, angiotensin II; Ang-(1-7), angiotensin-(1-7); AT1R, angiotensin two type one receptor; ERK1/2, extracellular regulated kinase one and two; HO-1, haem oxygenase one; JNK, c-Jun N-terminal kinases; MAPK, mitogen-activated protein kinase; Mas1, mas1 receptor; MMP-8, matrix metalloproteinase 8; NADPH, nicotinamide adenine dinucleotide phosphate; NFkB, nuclear factor kappa B; Nrf2, nuclear factor erythroid 2-related factor 2; PLC, phospholipase C; RhoA/ROCK2, RAAS homolog family member A/Rho associated coiled-coil containing protein kinase 2; ROS, reactive oxygen species; SHP-2, small heterodimer partner 2.

Ang-(1-7)-induced NO release by the post-translational regulation of eNOS (phosphorylation at Ser1177 and dephosphorylation at Thr495) is widely accepted as the primary event underlying vasoprotective effects of the peptide [1] (Figure 3). Wortmannin (a phosphatidylinositol 3-kinase (PI3K) inhibitor) blocked Ang-(1-7)-mediated activation of eNOS (Ser1177 phosphorylation) indicating that Ang-(1-7) regulates eNOS via Akt-dependent pathways. Ang-(1-7) also counter-regulates Ang II actions in endothelial cells by reducing Ang II-stimulated activation of c-Src, ERK1/2 and Nox by enhancing SHP-2 phosphorylation [73,74,95]. In human umbilical vein endothelial cells (HUVEC), Ang-(1-7) ameliorated Ang II-induced senescence by increasing the expression of the anti-ageing protein ‘Klotho’ and activation of the antioxidant, nuclear factor-erythroid-2-related factor (Nrf2)/haem-oxygenase 1 pathway [119]. It also blunted Ang II-induced expression of endoplasmic reticulum stress markers (p-eIF2α, ATF-6, CHOP, Grp78, ATF4) and amplified NO production to maintain endothelial cell function [120]. In human cerebral endothelial cells, Ang-(1-7) inhibited Ang II-stimulated ROS production, pro-apoptotic activity and reduced NO levels [121]. Furthermore, protective effects of Ang-(1-7) against other stress inducers, such as thrombin, have been demonstrated in endothelial cells [122].

In addition to regulating production of the vasodilator NO, Ang-(1-7) selectivity increases prostaglandin synthesis [123]. Chronic infusion of Ang-(1-7) in SHR increased urinary excretion of prostaglandin E_2_ and 6-keto-prostaglandin F_1α_ and induced diuresis, natriuresis, and decreased blood pressure [124]. In endothelial cells, Ang-(1-7) stimulates the production of vasodilator prostacyclins via activation of PLA2 and CaM kinase II/ MAP kinase [123], and in vascular smooth muscle cells prostacyclins play a role in Ang-(1-7) antiproliferative actions [123,125].

### Vascular smooth muscle cells and Ang-(1-7) signalling

Ang II is a potent vasoconstrictor that stimulates proliferation, inflammation, fibrosis and migration of vascular smooth muscle cells, processes involved in vascular remodelling in pathological conditions. It is now clear that Ang-(1-7) counteracts these actions by negatively regulating Ang II signaling [125–128]. In vascular smooth muscle cells, Ang-(1-7) opposes Ang II-stimulated pro-oxidative and proliferative signaling, reduces phosphorylation of c-Src, MAPKs and inhibits activation of Nox [105] (Figure 3). Ang-(1-7) attenuates Ang II-induced contraction by inhibiting RhoA/ROCK2 signaling and phosphorylation of moesin and myosin light chain [126]. Other signaling pathways influenced by Ang-(1-7) in vascular smooth muscle cells include NF-κB, IkBα, MAPK, and matrix metalloproteinases [127,128].

### Fibroblasts and Ang-(1-7) signalling

Studies in fibroblasts have also shown protective effects of Ang-(1-7). In renal interstitial fibroblasts, Ang-(1-7) significantly decreased aldosterone-induced pro-fibrotic signaling [129]. In cardiac fibroblasts from Sprague Dawley rats, Ang-(1-7)/Mas1 activated SHP-1, a redox-sensitive protein, and inhibited Ang II-induced phosphorylation of c-Src and its downstream signaling molecules ERK1/2, α-SMA and TGF-β [130]. Metabolomic studies in primary cardiac fibroblasts, demonstrated that Ang-(1-7) blunted Ang II-induced signaling by inhibiting calcium/calmodulin-dependent protein kinase II delta (CaMKIIδ), Nox4, CTGF and ERK1/2 [131].

## ACE2/Ang-(1-7) and vascular remodelling

Vascular remodelling is a complex process involving collagen deposition, fibrosis, inflammation, calcification and endothelial dysfunction and occurs with normal ageing and in response to injury and disease. In pathological conditions such as hypertension, atherosclerosis and diabetes mellitus, vascular remodelling is accelerated leading to target organ damage [132]. Studies in ACE2 knockout mice showed augmented vascular stiffness, worsening of vascular injury and potentiation of Ang II effects on oxidative stress, apoptosis and vascular smooth muscle cell phenotypic switching, while ACE2 overexpression inhibited vascular proliferation vascular hypertrophy and improved endothelium-dependent vasorelaxation [75,84,133,134]. Moreover, rhACE2 prevented Ang II-induced vascular remodelling through processes involving JAK-STAT-SOCS signaling [76]. Many of the vasoprotective effects of ACE2 were recapitulated by up-regulation of the Ang-(1-7)/Mas1 system, while pharmacological inhibition or gene deletion of Mas1 prevented ACE2/Ang-(1-7) beneficial effects [135]. In particular, in Mas1^−/−^ mice acetylcholine-induced vascular relaxation is blunted, and mice exhibit endothelial dysfunction, increased mean arterial blood pressure, cardiac dysfunction, reduced NO production, and increased oxidative stress [135,136]. There is thus convincing pre-clinical evidence that the ACE2/Ang-(1-7)/Mas1 axis negatively regulates Ang II and that it is vasoprotective, highlighting it as a potential therapeutic target in cardiovascular disease, especially in conditions associated with activation of the RAS.

## ACE2/Ang-(1-7)/Mas1 and vascular inflammation

Several studies have shown that the ACE2-Ang-(1-7)-Mas1 axis has anti-inflammatory properties in vascular injury [137]. *In vitro* studies in human aortic smooth muscle cells demonstrated that Ang-(1-7) counteracts inflammatory effects of Ang II by inhibiting iNOS, Nox and pro-inflammatory signaling pathways [138]. In human and rabbit endothelial cells, anti-inflammatory effects of Ang-(1-7) involve lectin-like oxidized low-density lipoprotein receptor-1 (LOX-1) [139]. *In vivo* studies support the anti-inflammatory role of Ang-(1-7). In a mouse model of atherosclerosis, chronic Ang-(1-7) treatment inhibited early atherosclerotic lesion formation by protecting endothelial function and inhibiting the inflammatory response [140].

## ACE2/Ang-(1-7)/Mas1, oxidative stress, antioxidants and vascular function

Many of the molecular processes that underlie vascular injury and inflammation in pathological conditions involve Ang II-induced activation of redox-sensitive pathways and oxidative stress [141]. The ACE2/Ang-(1-7)/Mas1 axis counter-regulates oxidative damage in the vascular system by reducing Nox-mediated ROS production and increasing antioxidant capacity [142,143]. In renal arteries from diabetic patients and in conduit and resistance arteries from diabetic mice activation of ACE2/Ang-(1-7) improved endothelial function by decreasing oxidative stress through processes that down-regulate Nox2 and up-regulate antioxidant enzymes [142]. Similar Nox2/ROS actions have been observed in Ang II-stimulated brain microvascular endothelial cells, where Ang-(1-7) opposed pro-apoptotic actions of Ang II by reducing Nox2 expression and decreasing ROS formation [121]. Other Nox isoforms, including Nox2 and Nox4, are also modulated by ACE2/Ang-(1-7) [144]. In a model of ischaemic stroke, Ang-(1-7)-induced neuroprotection was associated with decreased Nox1 expression [145]. In diabetic SHR, Ang-(1-7) treatment improved renal function by inhibiting the Nox4/ROS system [146] and in a mouse model of cardiac hypertrophy AVE 0991, a nonpeptide Ang-(1-7) analogue, prevented left ventricular hypertrophy and improved heart function by down-regulating Nox2 and Nox4 and decreasing oxidative stress [147]. In addition, ACE2 overexpression in endothelial progenitor cells decreased Nox4 expression and reduced ROS production [144]. These findings suggest that ACE2 activation may play a role in improving efficacy of endothelial progenitor cell-based therapy in vascular injury by blunting the Nox4/ROS system [144].

ACE2 and Ang-(1-7) further influence the redox state by regulating antioxidant enzymes, including superoxide dismutase (SOD) and Nrf2. Infusion of recombinant human ACE2 (GSK2586881) in patients with pulmonary hypertension was associated with increased plasma levels of SOD2 and reduced oxidative stress [91]. Similarly, in a mouse model of bleomycin-induced pulmonary hypertension, recombinant ACE2 treatment increased pulmonary SOD2 expression, reduced oxidative stress and attenuated development of vascular remodelling [148]. Protective effects of Ang-(1-7) in Ang II-induced cardiac hypertrophy involve Sirt-3 mediated deacetylation of Foxo3a, up-regulation of SOD2 and decreased ROS levels in cardiomyocytes [149]. ACE2/Ang-(1-7) also modulates the transcription factor Nrf2, the master regulator of antioxidant genes. Endothelial cell senescence is attenuated by Ang-(1-7) through pathways that involve Klotho and Nrf2 [119,150]. In models of hyperoxic lung injury, ACE2 activation inhibited inflammation, reduced oxidative stress and activated the Nrf2 antioxidant pathway [150].

Another mechanism whereby ACE2/Ang-(1-7) modulates the oxidative milieu involves a novel nuclear system. In renal cells Ang II-induced ROS production is attenuated by activation of nuclear ACE2/Ang-(1-7) [151]. The functional significance of the nuclear system remains unclear, but it may act as a protective mechanism to prevent accumulation of injurious ROS within the nucleus thereby preventing oxidative DNA damage [151].

## Interactions between ACE2/Ang-(1-7)/Mas1 and AT2 receptors

Similar to Mas1, activation of AT2R mediates vasoprotective effects by promoting vasodilation and inhibiting inflammation, fibrosis and apoptosis [152]. Growing evidence indicates that these G protein-coupled receptors interact, especially in the kidney, and that they heterodimerize [153–155]. AT2 and Mas1 receptors co-localize in the renal cortex of obese Zucker rats and are independently involved in the production of NO and the regulation of the natriuretic response [154]. In astrocytes, Mas1 and AT2R heterodimerize and are functionally dependent on each other [155]. The biological significance of Mas1:AT2R receptor dimerization remains unclear but interaction between these receptors may amplify signaling and vasoprotective properties.

In addition to physical links between Mas1 and AT2R, cross-talk between these receptors may be mediated by a common ligand, Ang-(1-7). This is supported by studies demonstrating that A779 as well as PD123319 (AT2R antagonist) prevent Ang-(1-7) cellular and vascular actions [153,156]. The inhibitory effects of A779 are well described. In mouse hearts pre-treated with the AT1R blocker losartan, PD123319 prevented Ang-(1-7) induced vasodilation and increased perfusion pressure [153]. Other studies demonstrated that PD123319 inhibits protective effects of Ang-(1-7) in models of endothelial dysfunction [157,158], hepatocellular carcinoma [159], atherosclerosis [160], ischaemic stroke [161], and cerebral autophagy in SHR [162]. However, some studies failed to show a role for AT2R in Ang-(1-7)-mediated effects supporting Mas1 as the primary receptor for the peptide [163,164].

Interactions between ACE2, Ang-(1-7), Mas1 and AT2R have pathophysiological significance. In experimental models of pulmonary hypertension, activation of the ACE2/Ang-(1-7)/Mas1 axis attenuates progression of disease, processes associated with upregulation of AT2R [165]. Further supporting this notion, chronic treatment with compound 21 (AT2R agonist) prevented cardiopulmonary fibrosis and attenuated pulmonary hypertension with an associated increase in mRNA expression of ACE2 and Mas1 indicating that beneficial effects of AT2R activation involve the ACE2-Ang-(1-7)-Mas1 pathway [166]. On the other hand, activation of ACE2/Ang-(1-7) increases expression of AT2R in models of SHR, diabetes, Ang II-induced hypertension and pulmonary hypertension [167–170].

## SARS-CoV-2 and COVID-19 implications of vascular ACE2

Beyond its vasoprotective functions, vascular ACE2 may play an important role in cardiovascular disease related to COVID-19, since ACE2 is the receptor through which SARS-CoV-2 enters host cells causing infection [20–23].

### ACE2 as the cell entry receptor for SARS-CoV-2

Viral infection studies in various ACE2 expressing cell types across species (human, bats, civet, pigs, mice), computational modelling and cryo-EM experiments demonstrated unambiguously that ACE2 is the receptor for SARS-CoV-2, similar to that for other coronaviruses including SARS-CoV and middle east respiratory syndrome coronavirus (MERS-CoV) [171]. Entry of SARS-CoV-2 into host cells depends on binding with the receptor via the virus surface spike proteins (S-proteins), of which there are 2: the S1-protein possesses a receptor-binding domain (RBD) responsible for viral binding to the host cell and the S2-protein, which contains a fusion peptide domain important for virus:host cell membrane fusion [172]. ACE2, as the cell entry receptor, is probably the key factor determining host cell tropism and infectivity. While ACE2 normally localizes on the plasma membrane with the extracellular N-terminal possessing the catalytic site for generation of Ang-(1-7) (and other peptides), it can undergo proteolytic shedding and post-translational modification by various proteases and enzymes, including transmembrane protease serine 2 (TMPRSS2), disintegrin and metalloproteinase domain-containing protein (ADAM)10, ADAM17 (also called TACE), cathepsins and furin [173,174]. Binding of SARS-CoV-2 via the S-protein to ACE2 prompts cleavage of ACE2 by ADAM17/TACE at the extracellular N-tail, which possesses catalytic activity, producing soluble ACE2 [26], while TMPRSS2 causes proteolytic cleavage of ACE2 at the intracellular C-domain without catalytic activity [23]. TMPRSS2, together with cathepsin B and L, primes ACE2 facilitating membrane fusion and internalization of the S protein:ACE2 complex, essential for host cell infection [23].

Numerous accessory proteins have been identified that may influence viral release, stability and virulence. The 3-terminus of the SARS-CoV-2 genome encodes eight accessory proteins (3a, 3b, p6, 7a, 7b, 8b, 9b and ORF14) which play important roles in the entry of SARS-CoV-2 [175]. SARS-CoV-2 S-protein 1 has also been shown to bind CD147 a transmembrane glycoprotein of the immunoglobulin superfamily [176], which was previously shown to facilitate SARS-CoV:host cell infection [177]. The complex regulatory processes of SARS-CoV-2:ACE2 interaction have been described in recent publications and the reader is referred to these for further details [21–23].

### SARS-CoV-2 and vascular ACE2

Vascular cells express many of the proteases involved in SARS-CoV-2-induced ACE2 post-translational modification, suggesting potential viral infection in the vasculature. ACE2 together with TMPRSS2, ADAM10, ADAM17 and cathepsins have been demonstrated in endothelial cells, although levels of ACE2 and TMPRSS2 are lower than in nasal epithelium [178]. Cardiovascular cells also express CD147 and may play a role in severity of COVID-19 disease in the elderly [178]. Despite increasing evidence that vascular ACE2 is involved in SARS-CoV-2-induced vascular injury, whether vascular cells are directly infected by the virus through endothelial or vascular smooth muscle cell ACE2 still needs to be unambiguously proven, especially in the clinical context.

Translating SARS-Cov-2:ACE2 experimental evidence to the clinic has been challenging. This may relate, in part, to the fact that murine and human ACE2 exhibit some differences with interspecies homology of 81.86% [179]. Both murine and human ACE2 possess collectrin and peptidase M2 domains but amino acid sequences differ in the domain to which the S1-protein binds. Amino acids Asp30, His34, Tyr41, Gln42, Lys353, Arg357, Gln24 and Met82 of human ACE2 play an important role in binding with viral S-protein [21]. Comparison of murine and human ACE2 sequences indicated that five out of these eight residues differ between the species [179]. These findings prompted the development of humanized ACE2 mice (which possess the human ACE2 amino acid sequence) for investigating SARS-CoV-2 pathology in the context of human disease [180].

### Vascular ACE2 and the pathophysiology of COVID-19-related cardiovascular disease

While COVID-19 is a viral disease of the respiratory system causing severe and progressive lung damage, it is also associated with significant cardiovascular comorbidities and sequelae [181–183]. In particular, hypertension and diabetes mellitus are major comorbidities of COVID-19, which itself predisposes to thromboembolic disease, acute myocardial injury, myocarditis, heart failure and arrhythmias [182,183]. Patients with COVID-19 who have pre-existing cardiovascular disease have more severe disease and worse outcomes than those without a history of cardiovascular disease [181,182,184,185]. The pathophysiological processes underlying COVID-19-related cardiovascular disease remain unclear, but the vascular RAS system probably plays an important role. In the lungs and other organs, vascular injury and loss of endothelial integrity cause tissue oedema, activation of coagulation pathways, inflammation, endotheliitis and vascular dysfunction, processes contributing to tissue damage and organ failure in COVID-19 [184,186,187].

**Figure 4:**
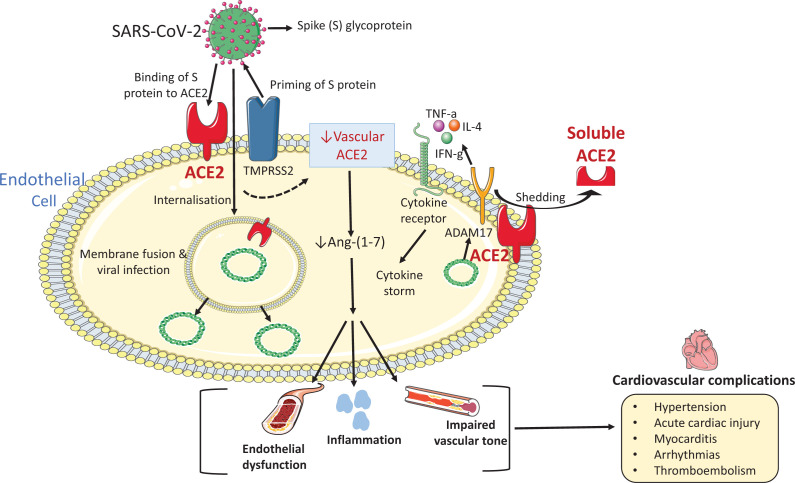
Putative role of vascular ACE2 in cardiovascular disease associated with COVID-19 Since vascular cells express ACE2, it may be possible that SARS-CoV-2 interaction with vascular ACE2 plays a role in cardiovascular disease associated with COVID-19. SARS-CoV-2 via its spike protein, which is primed by the endogenous transmembrane serine protease 2 (TMPRSS2), binds to ACE2 for cell entry. The virus is endocytosed leading to fusion of viral and cellular membranes and subsequent release of viral RNA into the cytosol which causes viral infection and the down-regulation of endogenous ACE2 and up-regulation of ADAM17. Following the vesicular transport to the cell surface, ADAM17 cleaves the extracellular domain of ACE2 and takes part in processing of different cytokines leading to activation of inflammatory signalling which further down-regulates vascular ACE2. Down-regulation in vascular ACE2 may lead to decreased Ang-(1-7) production contributing to endotheliitis, vascular inflammation and impaired vascular tone, which could promote cardiovascular complications in COVID-19. ACE2, angiotensin converting enzyme 2; ADAM17, tumour necrosis factor-alpha-converting enzyme; SARS-CoV-2, severe acute respiratory syndrome coronavirus 2.

At the molecular level, vascular ACE2 may be critical (Figure 4). Firstly, SARS-CoV-2 binding to vascular ACE2 may facilitate virus entry into endothelial and vascular smooth muscle cells causing viral infection of the endothelium and vascular media [188]. This probably involves other players as well, such as TMPRSS2 [23]. Post-mortem analysis of different organs in patients with COVID-19 demonstrated endothelial cell viral infection and endotheliitis [188]. Secondly, down-regulation of vascular ACE2 by SARS-CoV-2 may decrease ACE2 activity leading to reduced production of vasoprotective Ang-(1-7) causing endothelial dysfunction and increased vascular permeability [187]. SARS-CoV has been shown to downregulate myocardial ACE2 in patients with severe acute respiratory syndrome [189], and similar process may occur for SARS-CoV-2. Finally, viral infection and down-regulation of the ACE2-Ang-(1-7) pathway could promote increased generation of vascular-derived cytokines and vasoactive factors causing endotheliitis, vascular inflammation and impaired vascular tone [189,190]. Although these processes are plausible and vascular ACE2 might be pivotal in cardiovascular sequelae associated with COVID-19, this awaits confirmation.

### Targeting ACE2/Ang-(1-7)/Mas1 and AT2R as potential therapeutic approaches in COVID-19

Considering the importance of ACE2 in cardiovascular (patho)physiology, it has been suggested that the loss of ACE2 catalytic function and dysregulation of the RAS may be responsible for the cardiovascular symptoms reported in COVID-19 patients [181–183,186]. Accordingly, there is growing interest in targeting ACE2, as well as other RAS elements especially Ang-(1-7)/Mas1 and AT2R, as therapeutic strategies in COVID-19 [191]. Studies have suggested (i) ACE2 inhibitors to prevent viral entry, (ii) ACE2 activators to promote Ang-(1-7) anti-inflammatory actions and (iii) soluble human recombinant ACE2 (shrACE2) to saturate and inactivate circulating S-protein, as putative novel therapeutic modalities to prevent SARS-CoV-2 infection of host cells [191–195]. *In vitro* studies have shown that shrACE2 decreases SARS-CoV-2 infection and clinical trials using shrACE2 (APN01) are currently underway [194,195] (ClinicalTrials.gov identifier NCT04335136). Other clinical trials targeting Ang-(1-7) (ATCO trial) and Mas1 (ClinicalTrials.gov identifier NCT04332666; 04401423; 04375124) are also in progress. In addition, the AT2R agonist VP01 (C21) is being tested as a potential repurposed therapeutic in COVID-19 in the Angiotensin II Type Two Receptor Agonist Covid-19 Trial (ATTRACT) (ClinicalTrials.gov identifier NCT04452435). Outcomes from these clinical trials are awaited.

## Conclusions

Vascular function is tightly regulated by the RAS. While the molecular biology, biochemistry and physiology of Ang II are well defined, there is a paucity of information about the vascular ACE2/Ang-(1-7) system, especially in human health and disease. It is becoming increasingly apparent that ACE2, through Ang-(1-7)/Mas, is vasoprotective and that down-regulation of ACE2 promotes vascular injury and remodelling, processes that are ameliorated when ACE2 or Ang-(1-7) are up-regulated. Vascular ACE2 is controlled by humoral and mechanical factors and upon ADAM17 activation is shed from endothelial cells into the plasma, where circulating soluble ACE2 might be a predictive marker of vascular health or disease. ACE2 is also regulated by drugs that inhibit the RAS. ACEi and ARBs increase expression of ACE2 and may influence soluble ACE2 activity. Beyond its vasoprotective role in the cardiovascular system, ACE2 is crucially involved in the pathophysiology of COVID-19 as the receptor for SARS-CoV-2. COVID-19 is increasingly being considered as an endothelial and vascular disease. Considering the importance of ACE2 in endothelial and vascular cells, we speculate that vascular ACE2 may play a role in the cardiovascular risk factors and complications of COVID-19. However, this awaits confirmation. Vascular ACE2 is emerging as a major regulatory system potentially involved in many pathologies. It is therefore not surprising that the ACE2/Ang-(1-7)/Mas1 system is gaining enormous interest as a therapeutic target.

## Abbreviations

α-SMA: alpha-smooth muscle actin
ARB: angiotensin II receptor blocker
ATF-4: activating transcription factor-4
ATF-6: activating transcription factor-6
CHOP: CCAAT-enhancer-binding protein homologous protein
cPLA_2_: cyclic phospholipase A2
c-SRC: SRC proto-oncogene, non-receptor tyrosine kinase
CTGF: connective tissue growth factor
eIF2α: eukaryotic translation initiation factor 2A
ERK: extracellular signal regulated kinase
Grp78: glucose regulated protein, 78kD
IκBα: I-kappa-B-alpha
JNK: c-Jun N-terminal kinases
NADPH: nicotinamide adenine dinucleotide phosphate
NFκB: nuclear factor kappa B
Nox: NADPH oxidase
Nrf2: nuclear factor erythroid 2-related factor 2
PKCβII: protein kinase C βII
RhoA/ROCK2: RAAS homolog family member A/Rho associated coiled-coil containing protein kinase 2
RNA: ribonucleic acid
Ser: serine
SHP: small heterodimer partner
TGF-β: transforming growth factor beta
Thr: threonine

## References

[1] Santos R., Sampaio W., Alzamora A., Motta-Santos D., Alenina N. and Bader M. (2018) The ACE2/Angiotensin-(1-7)/MAS1 axis of the Renin-angiotensin system: Focus on angiotensin-(1-7). Physiol. Rev. 98, 505–553 10.1152/physrev.00023.2016 29351514 PMC7203574

[2] Bader M. and Ganten D (2008) Update on tissue renin-angiotensin systems. J. Mol. Med. 86, 615–621 10.1007/s00109-008-0336-0 18414822

[3] Moon J. (2013) Recent update of Renin-angiotensin-aldosterone system in the pathogenesis of hypertension. Electrolyte. Blood. Press. 11, 41 10.5049/EBP.2013.11.2.41 24627703 PMC3950224

[4] Tipnis S., Hooper N., Hyde R., Karran E., Christie G. and Turner A. (2000) A human homolog of Angiotensin-converting enzyme. J. Biol. Chem. 275, 33238–33243 10.1074/jbc.M002615200 10924499

[5] Turner A., Tipnis S., Guy J., Rice G. and Hooper N. (2002) ACEH/ACE2 is a novel mammalian metallocarboxypeptidase and a homologue of angiotensin-converting enzyme insensitive to ACE inhibitors*. Can*. J. Physiol. Pharmacol. 80, 346–353 10.1139/y02-021 12025971

[6] Santos R.A.S, Oudit G.Y., Verano-Braga T., Canta G., Steckelings U.M. and Bader M (2019) The renin-angiotensin system: going beyond the classical paradigms. Am. J. Physiol. Heart Circ. Physiol. 316, 958–970 10.1152/ajpheart.00723.2018 PMC7191626 30707614

[7] Kaschina E. and Unger T. (2003) Angiotensin AT1/AT2 receptors: Regulation, Signaling and Function. Blood Press 12, 70–88 10.1080/08037050310001057 12797627

[8] Forrester S.J., Booz G.W., Sigmund C.D., Coffman T.M., Kawai T. and Rizzo V. (2018) Angiotensin II signal transduction: An update on mechanisms of physiology and pathophysiology. Physiol. Rev. 98, 1627–1738 10.1152/physrev.00038.2017 29873596 PMC6335102

[9] Lemarié C. and Schiffrin E. (2009) The angiotensin II type 2 receptor in cardiovascular disease. J. Renin Angiotensin Aldosterone Syst. 11, 19–31 10.1177/1470320309347785 19861349

[10] Nguyen D.C.A. and Touyz R (2011) A new look at the renin-angiotensin system— Focusing on the vascular system. Peptides 32, 2141–2150 10.1016/j.peptides.2011.09.010 21945916

[11] Savoia C., Burger D., Nishigaki N., Montezano A. and Touyz R. (2011) Angiotensin II and the vascular phenotype in hypertension. Expert Rev. Mol. Med. 13, e11 10.1017/S1462399411001815 21450123

[12] Donoghue M., Hsieh F., Baronas E., Godbout K., Gosselin M., Stagliano N. et al. (2000) Novel angiotensin-converting enzyme-related carboxypeptidase (ACE2) converts Angiotensin I to Angiotensin 1-9. Circ. Res. 87, 5 10.1161/01.RES.87.5.e1 10969042

[13] Mendoza-Torres E., Oyarzún A., Mondaca-Ruff D., Azocar A. and Castro P.F. Jalil J.E. et al. (2015) ACE2 and vasoactive peptides: novel players in cardiovascular/renal remodelling and hypertension. Ther. Adv. Cardiovasc Dis. 9, 217–237 10.1177/1753944715597623 26275770

[14] Danilczyk U. and Penninger J. (2006) Angiotensin-Converting Enzyme II in the heart and the kidney. Cir. Res. 98, 463–471 10.1161/01.RES.0000205761.22353.5f 16514079

[15] Reudelhuber T. (2006) A place in our hearts for the lowly Angiotensin 1-7 peptide? Hypertension 47, 811–815 10.1161/01.HYP.0000209020.69734.73 16520407

[16] Santos R. (2014) Angiotensin-(1-7). Hypertension 63, 1138–1147 10.1161/HYPERTENSIONAHA.113.01274 24664288

[17] Pereira M., Souza L., Becari C., Duarte D., Camacho F., Oliveira J. et al. (2013) Angiotensin II-Independent Angiotensin-(1-7) formation in rat hippocampus. Hypertension 62, 879–885 10.1161/HYPERTENSIONAHA.113.01613 24041943

[18] Santos R., e Silva A., Maric C., Silva D., Machado R., de Buhr I. et al. (2003) Angiotensin-(1-7) is an endogenous ligand for the G protein-coupled receptor Mas. Proc. Natl. Acad. Sci. U. S. A. 100, 8258–8263 10.1073/pnas.1432869100 12829792 PMC166216

[19] Tetzner A., Gebolys K., Meinert C., Klein S., Uhlich A., Trebicka J. et al. (2016) G-Protein-Coupled Receptor MrgD is a receptor for Angiotensin-(1-7) involving Adenylyl Cyclase, cAMP, and Phosphokinase A. Hypertension 68, 185–194 10.1161/HYPERTENSIONAHA.116.07572 27217404

[20] Gheblawi M., Wang K., Viveiros A., Nguyen Q., Zhong J.C., Turner A.J. et al. (2020) Angiotensin-Converting Enzyme 2: SARS- CoV-2 receptor and regulator of the Renin-Angiotensin System: Celebrating the 20th anniversary of the discovery of ACE2. Circ. Res. 126, 1456–1474 10.1161/CIRCRESAHA.120.317015 32264791 PMC7188049

[21] Yan R., Zhang Y., Li Y., Xia L., Guo Y. and Zhou Q. (2020) Structural basis for the recognition of SARS-CoV-2 by full-length human ACE2. Science 367, 1444–1448 10.1126/science.abb2762 32132184 PMC7164635

[22] Wan Y., Shang J., Graham R., Baric R. and Li F. (2020) Receptor recognition by the novel coronavirus from Wuhan: an analysis based on decade-long structural studies of SARS coronavirus. J. Virol. 94, 7 10.1128/JVI.00127-20 PMC7081895 31996437

[23] Hoffmann M., Kleine-Weber H., Schroeder S., Krüger N., Herrler T., Erichsen S. et al. (2020) SARS-CoV-2 cell entry depends on ACE2 and TMPRSS2 and is blocked by a clinically proven protease inhibitor. Cell 181, 271–280 10.1016/j.cell.2020.02.052 32142651 PMC7102627

[24] Crackower M., Sarao R., Oudit G., Yagil C., Kozieradzki I., Scanga S. et al. (2002) Angiotensin-converting enzyme 2 is an essential regulator of heart function. Nature 417, 822–828 10.1038/nature00786 12075344

[25] Rice G., Thomas D. and Grant P. (2020) Evaluation of angiotensin-converting enzyme (ACE), its homologue ACE2 and neprilysin in angiotensin peptide metabolism. Biochem. J. 383, 45–51 10.1042/BJ20040634 PMC1134042 15283675

[26] Lambert D., Yarski M., Warner F., Thornhill P., Parkin E., Smith A. et al. (2005) Tumor Necrosis Factor-α Convertase (ADAM17) mediates regulated ectodomain shedding of the severe-acute respiratory syndrome-coronavirus (SARS- CoV) receptor, Angiotensin-converting Enzyme-2 (ACE2). J. Biol. Chem. 280, 30113–30119 10.1074/jbc.M505111200 15983030 PMC8062222

[27] Wysocki J., Ye M., Rodriguez E., González-Pacheco F., Barrios C., Evora K. et al. (2010) Targeting the degradation of Angiotensin II with recombinant angiotensin- converting enzyme 2. Hypertension 55, 90–98 10.1161/HYPERTENSIONAHA.109.138420 19948988 PMC2827767

[28] Patel S.K., Velkoska E., Freeman M., Wai B., Lancefield T.F. and Burrell L.M. (2014) From gene to protein-experimental and clinical studies of ACE2 in blood pressure control and arterial hypertension. Front. Physiol. 5, 227–236 10.3389/fphys.2014.00227 25009501 PMC4067757

[29] Lew R., Warner F., Hanchapola I., Yarski M., Manohar J., Burrell L. et al. (2008) Angiotensin-converting enzyme 2 catalytic activity in human plasma is masked by an endogenous inhibitor. Exp. Phys. 93, 685–693 10.1113/expphysiol.2007.040352 PMC7197901 18223027

[30] Epelman S., Tang W., Chen S., Van Lente F., Francis G. and Sen S. (2008) Detection of soluble angiotensin-converting enzyme 2 in heart failure. Insights into the endogenous counter-regulatory pathway of the Renin-angiotensin-aldosterone system. J. Am. Coll. Cardiol. 102, 2–4 10.1016/j.jacc.2008.02.088 PMC2856943 18718423

[31] Yan T., Xiao R. and Lin G. (2020) Angiotensin-converting enzyme 2 in severe acute respiratory syndrome coronavirus and SARS-CoV-2: A double-edged sword? FASEB. J. 34, 6017–6026 10.1096/fj.202000782 32306452 PMC7264803

[32] Sama I., Ravera A., Santema B., van Goor H., ter Maaten J., Cleland J. et al. (2020) Circulating plasma concentrations of angiotensin-converting enzyme 2 in men and women with heart failure and effects of renin-angiotensin-aldosterone inhibitors. Eur. Heart J. 41, 1810–1817 10.1093/eurheartj/ehaa373 32388565 PMC7239195

[33] Perlot T. and Penninger J.M. (2013) ACE2 - from the renin-angiotensin system to gut microbiota and malnutrition. Microbes Infect 15, 866–873 10.1016/j.micinf.2013.08.003 23962453 PMC7110844

[34] Lai Z.W., Lew R.A., Yarski M.A., Mu F.T., Andrews R.K. and Smith A.I. (2009) The identification of a calmodulin-binding domain within the cytoplasmic tail of angiotensin-converting enzyme-2. Endocrinology 150, 2376–2381 10.1210/en.2008-1274 19164471 PMC7108506

[35] Lambert D.W., Clarke N.E., Hooper N.M. and Turner A.J. (2008) Calmodulin interacts with angiotensin-converting enzyme-2 (ACE2) and inhibits shedding of its ectodomain. FEBS. Lett. 582, 385–390 10.1016/j.febslet.2007.11.085 18070603 PMC7094239

[36] Sluimer J., Gasc J., Hamming I., van Goor H., Michaud A., van den Akker L. et al. (2008) Angiotensin-converting enzyme 2 (ACE2) expression and activity in human carotid atherosclerotic lesions. J. Pathol. 215, 273–279 10.1002/path.2357 18498093

[37] Hamming I., Timens W., Bulthuis M., Lely A., Navis G. and van Goor H. (2004) Tissue distribution of ACE2 protein, the functional receptor for SARS coronavirus. A first step in understanding SARS pathogenesis. J. Pathol. 203, 631–637 10.1002/path.1570 15141377 PMC7167720

[38] Burrell L., Risvanis J., Kubota E., Dean R., MacDonald P., Lu S. et al. (2005) Myocardial infarction increases ACE2 expression in rat and humans. Eur. Heart J. 26, 369–375 10.1093/eurheartj/ehi114 15671045

[39] Hikmet F., Méar L., Edvinsson Å., Micke P., Uhlén M. and Lindskog C. (2020) The protein expression profile of ACE2 in human tissues. Mol. Sys. Biol. 16, e9610 10.15252/msb.20209610 PMC7383091 32715618

[40] Valdés G., Neves L., Anton L., Corthorn J., Chacón C., Germain A. et al. (2006) Distribution of Angiotensin-(1-7) and ACE2 in human placentas of normal and pathological pregnancies. Placenta 27, 200–207 10.1016/j.placenta.2005.02.015 16338465

[41] Zhang J., Dong J., Martin M., He M., Gongol B., Marin T. et al. (2018) AMP-activated protein kinase phosphorylation of Angiotensin-Converting Enzyme 2 in endothelium mitigates pulmonary hypertension. Am. J. Respir. Crit. Care Med. 198, 509–520 10.1164/rccm.201712-2570OC 29570986 PMC6118028

[42] Zulli A., Burrell L.M., Buxton B.F. and Hare D.L. (2008) ACE2 and AT4R are present in diseased human blood vessels. Eur. J. Histochem. 52, 39–44 10.4081/1184 18502721

[43] Prasad T., Verma A. and Li Q. (2020) Expression and cellular localization of the Mas receptor in the adult and developing mouse retina. Mol. Vis. 17, 1443–1455 PMC4203581 25352750

[44] Huang W., Cao Y., Liu Y., Ping F., Shang J., Zhang Z. et al. (2018) Activating Mas receptor protects human pulmonary microvascular endothelial cells against LPS-induced apoptosis via the NF-kB p65/P53 feedback pathways. J. Cell. Physiol. 234, 12865–12875 10.1002/jcp.27951 30537127 PMC13483338

[45] Kumar M., Grammas P., Giacomelli F. and Wiener J. (1996) Selective expression of c-mas proto-oncogene in rat cerebral endothelial cells. J. Mol. Neurosci. 8, 93–96 10.1097/00001756-199612200-00019 9051759

[46] Nicin L., Abplanalp W.T., Mellentin H., Kattih B., Tombor L., John D. et al. (2020) Cell type-specific expression of the putative SARS-CoV-2 receptor ACE2 in human hearts. Eur. Heart J. 41, 1804–1806 10.1093/eurheartj/ehaa311 32293672 PMC7184464

[47] Ramchand J., Patel S., Srivastava P., Farouque O. and Burrell L. (2018) Elevated plasma angiotensin converting enzyme 2 activity is an independent predictor of major adverse cardiac events in patients with obstructive coronary artery disease. Plos One 13, e0198144 10.1371/journal.pone.0198144 29897923 PMC5999069

[48] Haschke M., Schuster M., Poglitsch M., Loibner H., Salzberg M. and Bruggisser M. (2013) Pharmacokinetics and pharmacodynamics of recombinant human angiotensin- converting enzyme 2 in healthy human subjects. Clin. Pharmacokinet. 52, 783–792 10.1007/s40262-013-0072-7 23681967

[49] Lambert D.W., Lambert L.A., Clarke N.E., Hooper N.M., Porter K.E. and Turner A.J. (2014) Angiotensin converting enzyme 2 is subject to post-transcriptional regulation by miR-421. Clin. Sci. (Lond.) 127, 243–249 10.1042/CS20130420 24564768

[50] Tie Y., Zhai C., Zhang Y., Qin X., Yu F., Li H. et al. (2018) CCAAT/enhancer-binding protein β overexpression alleviates myocardial remodelling by regulating angiotensin-converting enzyme-2 expression in diabetes. J. Cell. Mol. Med. 22, 1475–1488 10.1111/jcmm.13406 29266779 PMC5824391

[51] Clarke N.E., Belyaev N.D., Lambert D.W. and Turner A.J. (2014) Epigenetic regulation of angiotensin-converting enzyme 2 (ACE2) by SIRT1 under conditions of cell energy stress. Clin. Sci. (Lond.) 126, 507–516 10.1042/CS20130291 24147777

[52] Koka V., Huang X.R., Chung A.C., Wang W., Truong L.D. and Lan H.Y. (2008) Angiotensin II up-regulates angiotensin I-converting enzyme (ACE), but down-reg-ulates ACE2 via the AT1-ERK/p38 MAP kinase pathway. Am. J. Pathol. 172, 1174–1183 10.2353/ajpath.2008.070762 18403595 PMC2329828

[53] Zhu L., Carretero O.A., Xu J., Harding P., Ramadurai N., Gu X. et al. (2015) Activation of angiotensin II type 2 receptor suppresses TNF-α-induced ICAM-1 via NF-кB: possible role of ACE2. Am. J. Physiol. Heart Circ. Physiol. 309, H827–H883 10.1152/ajpheart.00814.2014 PMC4591403 26163449

[54] Young M.J., Clyne C.D. and Chapman K.E. (2020) Endocrine aspects of ACE2 regulation: RAAS, steroid hormones and SARS-CoV-2. J. Endocrinol. 247, R45–R62 10.1530/JOE-20-0260 32966970

[55] Yamamuro M., Yoshimura M., Nakayama M., Abe K., Sumida H. and Sugiyama S. (2008) Aldosterone, but not angiotensin II, reduces angiotensin converting enzyme 2 gene expression levels in cultured neonatal rat cardiomyocytes. Circ. J. 72, 1346–1350 10.1253/circj.72.1346 18654024

[56] Fukuda S., Horimai C., Harada K., Wakamatsu T., Fukasawa H., Muto S. et al. (2011) Aldosterone-induced kidney injury is mediated by NFκB activation. J. Clin. Exp. Nephrol. 15, 41–49 10.1007/s10157-010-0373-1 PMC7087855 21072674

[57] Song J., Qu H., Hu B., Bi C., Li M., Wang L. et al. (2020) Physiological cyclic stretch up-regulates angiotensin-converting enzyme 2 expression to reduce proliferation and migration of vascular smooth muscle cells. Biosci. Rep. 40, BSR20192012 10.1042/BSR20192012 32463098 PMC7295630

[58] Kai H. and Kai M. (2020) Interactions of coronaviruses with ACE2, angiotensin II, and RAS inhibitors—lessons from available evidence and insights into COVID-19. Hypertens. Res. 43, 648–654 10.1038/s41440-020-0455-8 32341442 PMC7184165

[59] Ferrario C., Jessup J., Chappell M., Averill D., Brosnihan K., Tallant E. et al. (2005) Effect of angiotensin-converting enzyme inhibition and angiotensin II receptor blockers on cardiac angiotensin-converting enzyme 2. Circulation 111, 2605–2610 10.1161/CIRCULATIONAHA.104.510461 15897343

[60] Campbell D., Zeitz C., Esler M. and Horowitz J. (2004) Evidence against a major role for angiotensin converting enzyme-related carboxypeptidase (ACE2) in angiotensin peptide metabolism in the human coronary circulation. J. Hypertens. 22, 1971–1976 10.1097/00004872-200410000-00020 15361769

[61] Epelman S., Shrestha K., Troughton R., Francis G., Sen S., Klein A. et al. (2009) Soluble Angiotensin-Converting Enzyme 2 in Human Heart Failure: Relation With Myocardial Function and Clinical Outcomes. J. Card. Fail. 15, 565–557 10.1016/j.cardfail.2009.01.014 19700132 PMC3179261

[62] Walters T., Kalman J., Patel S., Mearns M., Velkoska E. and Burrell L. (2017) Angiotensin converting enzyme 2 activity and human atrial fibrillation: increased plasma angiotensin converting enzyme 2 activity is associated with atrial fibrillation and more advanced left atrial structural remodelling. Europace 19, 1280–1287 27738071 10.1093/europace/euw246

[63] Ramchand J., Patel S., Kearney L., Matalanis G., Farouque O., Srivastava P. et al. (2020) Plasma ACE2 activity predicts mortality in aortic stenosis and is associated with severe myocardial fibrosis. JACC Cardiovasc. Imaging 13, 655–666 10.1016/j.jcmg.2019.09.005 31607667

[64] Furuhashi M., Moniwa N., Mita T., Fuseya T., Ishimura S., Ohno K. et al. (2014) Urinary angiotensin-converting enzyme 2 in hypertensive patients may be increased by olmesartan, an angiotensin II receptor blocker. Am. J. Hypertens. 28, 15–21 10.1093/ajh/hpu086 24842388

[65] Wang X., Ye Y., Gong H., Wu J., Yuan J., Wang S. et al. (2016) The effects of different angiotensin II type 1 receptor blockers on the regulation of the ACE-AngII-AT1 and ACE2-Ang(1-7)-Mas axes in pressure overload-induced cardiac remodeling in male mice. J. Mol. Cell. Cardiol. 97, 180–190 10.1016/j.yjmcc.2016.05.012 27210827

[66] Ocaranza M., Godoy I., Jalil J., Varas M., Collantes P., Pinto M. et al. (2006) Enalapril attenuates downregulation of angiotensin-converting enzyme 2 in the late phase of ventricular dysfunction in myocardial infarcted rat. Hypertension 48, 572–578 10.1161/01.HYP.0000237862.94083.45 16908757

[67] Burchill L., Velkoska E., Dean R., Griggs K., Patel S. and Burrell L. (2012) Combination renin-angiotensin system blockade and angiotensin-converting enzyme 2 in experimental myocardial infarction: implications for future therapeutic directions. Clin. Sci. (Lond.) 123, 649–658 10.1042/CS20120162 22715807

[68] Yang Z., Yu X., Cheng L., Miao L., Li H., Han L. et al. (2013) Effects of enalapril on the expression of cardiac angiotensin-converting enzyme and angiotensin-converting enzyme 2 in spontaneously hypertensive rats. Arch. Cardiovasc. Dis. 106, 196–201 10.1016/j.acvd.2013.01.004 23706365

[69] Igase M., Strawn W., Gallagher P., Geary R. and Ferrario C. (2005) Angiotensin II AT1 receptors regulate ACE2 and angiotensin-(1-7) expression in the aorta of spontaneously hypertensive rats. Am. J. Physiol. Heart Circ. Physiol. 289, 1013–1019 10.1152/ajpheart.00068.2005 15833808

[70] Ishiyama Y., Gallagher P., Averill D., Tallant E., Brosnihan K. and Ferrario C. (2004) Upregulation of angiotensin-converting enzyme 2 after myocardial infarction by blockade of angiotensin II receptors. Hypertension 43, 970–976 10.1161/01.HYP.0000124667.34652.1a 15007027

[71] Iyer S., Ferrario C. and Chappell M. (1998) Angiotensin-(1-7) contributes to the antihypertensive effects of blockade of the renin-angiotensin system. Hypertension 31, 356–361 10.1161/01.HYP.31.1.356 9453328

[72] Keidar S., Gamliel-Lazarovich A., Kaplan M., Pavlotzky E., Hamoud S., Hayek T. et al. (2005) Mineralocorticoid receptor blocker increases angiotensin-converting enzyme 2 activity in congestive heart failure patients. Cir. Res. 97, 946–953 10.1161/01.RES.0000187500.24964.7A 16179584

[73] Sampaio W.O., Henrique de Castro C., Santos R.A., Schiffrin E.L. and Touyz R.M. (2007) Angiotensin-(1-7) counterregulates angiotensin II signaling in human endothelial cells. Hypertension 50, 1093–1098 10.1161/HYPERTENSIONAHA.106.084848 17984366

[74] Sampaio W., Souza dos Santos R., Faria-Silva R., da Mata Machado L., Schiffrin E. and Touyz R. (2007) Angiotensin-(1-7) through receptor mas mediates endothelial nitric oxide synthase activation via akt-dependent pathways. Hypertension 49, 185–192 10.1161/01.HYP.0000251865.35728.2f 17116756

[75] Sahara M., Ikutomi M., Morita T., Minami Y., Nakajima T., Hirata Y. et al. (2013) Deletion of angiotensin-converting enzyme 2 promotes the development of atherosclerosis and arterial neointima formation. Cardiovasc. Res. 101, 236–246 10.1093/cvr/cvt245 24193738

[76] Song B., Jin H., Yu X., Zhang Z., Yu H., Ye J. et al. (2013) Angiotensin- converting enzyme 2 attenuates oxidative stress and VSMC proliferation via the JAK2/STAT3/SOCS3 and profilin-1/MAPK signaling pathways. Regul. Pept. 185, 44–51 10.1016/j.regpep.2013.06.007 23816468

[77] Zhang C., Zhao Y.X., Zhang Y.H., Zhu L., Deng B.P., Zhou Z.L. et al. (2010) Angiotensin-converting enzyme 2 attenuates atherosclerotic lesions by targeting vascular cells. Proc. Natl. Acad. Sci. U. S. A. 107, 15886–15891 10.1073/pnas.1001253107 20798044 PMC2936602

[78] Wang J., Li J., Cheng C. and Liu S. (2020) Angiotensin-converting enzyme 2 augments the effects of endothelial progenitor cells-exosomes on vascular smooth muscle cell phenotype transition. Cell Tissue Res 382, 509–518 10.1007/s00441-020-03259-w 32852610

[79] Rentzsch B., Todiras M., Iliescu R., Popova E., Campos L.A., Oliveira M.L. et al. (2008) Transgenic angiotensin-converting enzyme 2 overexpression in vessels of SHRSP rats reduces blood pressure and improves endothelial function. Hypertension 52, 967–973 10.1161/HYPERTENSIONAHA.108.114322 18809792

[80] Díez-Freire C., Vázquez J., Correa de Adjounian M.F., Ferrari M.F., Yuan L., Silver X. et al. (2006) ACE2 gene transfer attenuates hypertension- linked pathophysiological changes in the SHR. Physiol. Genomics 27, 12–19 10.1152/physiolgenomics.00312.2005 16788004

[81] Hernández Prada J., Ferreira A., Katovich M., Shenoy V., Qi Y., Santos R. et al. (2008) Structure-based identification of small-molecule angiotensin-converting enzyme 2 activators as novel antihypertensive agents. Hypertension 51, 1312–1317 10.1161/HYPERTENSIONAHA.107.108944 18391097

[82] Lo J., Patel V.B., Wang Z., Levasseur J., Kaufman S., Penninger J.M. et al. (2013) Angiotensin-converting enzyme 2 antagonizes angiotensin II-induced pressor response and NADPH oxidase activation in Wistar-Kyoto rats and spontaneously hypertensive rats. Exp. Physiol. 98, 109–122 10.1113/expphysiol.2012.067165 22750422

[83] Rabelo L., Todiras M., Nunes-Souza V., Qadri F., Szijártó I., Gollasch M. et al. (2016) Genetic deletion of ace2 induces vascular dysfunction in c57bl/6 mice: role of nitric oxide imbalance and oxidative stress. Plos One 11, e015025 10.1371/journal.pone.0150255 PMC4829150 27070147

[84] Thomas M., Pickering R., Tsorotes D., Koitka A., Sheehy K., Bernardi S et al. (2010) Genetic Ace2 Deficiency Accentuates Vascular Inflammation and Atherosclerosis in the ApoE Knockout Mouse. Circ. Res. 107, 888–897 10.1161/CIRCRESAHA.110.219279 20671240

[85] Zhang C., Zhao Y.X., Zhang Y.H., Zhu L., Deng B.P., Zhou Z.L. et al. (2010) Angiotensin-converting enzyme 2 attenuates atherosclerotic lesions by targeting vascular cells. Proc. Natl. Acad. Sci. U. S. A. 107, 15886–15891 10.1073/pnas.1001253107 20798044 PMC2936602

[86] Shenoy V., Kwon K., Rathinasabapathy A., Lin S., Jin G., Song C. et al. (2014) Oral delivery of angiotensin-converting enzyme 2 and angiotensin-(1-7) bioencapsulated in plant cells attenuates pulmonary hypertension. Hypertension 64, 1248–1259 10.1161/HYPERTENSIONAHA.114.03871 25225206 PMC4239698

[87] Ferreira A., Shenoy V., Yamazato Y., Sriramula S., Francis J., Yuan L. et al. (2009) Evidence for angiotensin-converting enzyme 2 as a therapeutic target for the prevention of pulmonary hypertension. Am. J. Respir. Crit. Care Med. 179, 1048–1054 10.1164/rccm.200811-1678OC 19246717 PMC2689912

[88] Patel V.B., Zhong J.C., Fan D., Basu R., Morton J.S., Parajuli N. et al. (2014) Angiotensin-converting enzyme 2 is a critical determinant of angiotensin II-induced loss of vascular smooth muscle cells and adverse vascular remodelling. Hypertension 64, 157–164 10.1161/HYPERTENSIONAHA.114.03388 24799609

[89] Thatcher S.E., Zhang X., Howatt D.A., Yiannikouris F., Gurley S.B., Ennis T. et al. (2014) Angiotensin-converting enzyme 2 decreases formation and severity of angiotensin II-induced abdominal aortic aneurysms. Arterioscler. Thromb. Vasc. Biol. 34, 2617–2623 10.1161/ATVBAHA.114.304613 25301841 PMC4250973

[90] Chirinos J.A., Cohen J.B., Zhao L., Hanff T., Sweitzer N., Fang J. et al. (2020) Clinical and proteomic correlates of plasma ACE2 (angiotensin-converting enzyme 2) in human heart failure. Hypertension 76, 1526–1536 10.1161/HYPERTENSIONAHA.120.15829 32981365 PMC10681288

[91] Hemnes A.R., Rathinasabapathy A., Austin E.A., Brittain E.L., Carrier E.J., Chen X. et al. (2018) A potential therapeutic role for angiotensin-converting enzyme 2 in human pulmonary arterial hypertension. Eur. Respir. J. 51, 1702638 10.1183/13993003.02638-2017 29903860 PMC6613216

[92] Khan A., Benthin C., Zeno B., Albertson T., Boyd J., Christie J. et al. (2017) A pilot clinical trial of recombinant human angiotensin-converting enzyme 2 in acute respiratory distress syndrome. Crit. Care 21, 234 10.1186/s13054-017-1823-x 28877748 PMC5588692

[93] Joshi S., Wollenzien H., Leclerc E. and Jarajapu Y. (2019) Hypoxic regulation of angiotensin-converting enzyme 2 and Mas receptor in human CD34+ cells. J. Cell. Physiol. 234, 20420–20431 10.1002/jcp.28643 30989646 PMC6660366

[94] Joshi S., Mahoney S., Jahan J., Pitts L., Hackney K. and Jarajapu Y. (2020) Blood flow restriction exercise stimulates mobilization of hematopoietic stem/progenitor cells and increases the circulating ACE2 levels in healthy adults. J. Appl. Physiol. 128, 1423–1431 10.1152/japplphysiol.00109.2020 32324479 PMC7272753

[95] Touyz R. and Montezano A. (2018) Angiotensin-(1-7) and vascular function. Hypertension 71, 68–69 10.1161/HYPERTENSIONAHA.117.10406 29203630

[96] Van Twist D.J., Houben A.J., de Haan M.W., Mostard G.J., Kroon A.A. and de Leeuw P.W. (2013) Angiotensin-(1-7)-induced renal vasodilation in hypertensive humans is attenuated by low sodium intake and angiotensin II co-infusion. Hypertension 62, 789–793 10.1161/HYPERTENSIONAHA.113.01814 23918750

[97] de Moraes P.L., Kangussu L.M., Castro C.H., Almeida A.P., Santos R.A.S. and Ferreira A.J. (2017) Vasodilator effect of angiotensin-(1-7) on vascular coronary bed of rats: role of Mas, ACE and ACE2. Protein Pept. Lett. 24, 869–875 10.2174/0929866524666170728154459 28758595

[98] Fernandes L., Fortes Z.B., Nigro D., Tostes R.C.A., Santos R.A.S. and Carvalho M.H.C (2001) Potentiation of bradykinin by angiotensin-(1-7) on arterioles of spontaneously hypertensive rats studies in vivo. Hypertension 37, 703–709 10.1161/01.HYP.37.2.703 11230360

[99] Wilsdorf T., Gainer J.V., Murphey L.J., Vaughan D.E. and Brown N.J. (2001) Angiotensin-(1-7) does not affect vasodilator or TPA responses to bradykinin in human forearm. Hypertension 37, 1136–1140 10.1161/01.HYP.37.4.1136 11304515

[100] Sasaki S., Higashi Y., Nakagawa K., Matsuura H., Kajiyama G. and Oshima T. (2001) Effects of Angiotensin-(1-7) on forearm circulation in normotensive subjects and patients with essential hypertension. Hypertension 38, 90–94 10.1161/01.HYP.38.1.90 11463766

[101] Ueda S., Masumori-Maemoto S., Wada A., Ishii M., Brosnihan K. and Umemura S. (2001) Angiotensin (1-7) potentiates bradykinin-induced vasodilatation in man. J. Hypertens. 19, 2001–2009 10.1097/00004872-200111000-00010 11677365

[102] Ueda S., Masumori-Maemoto S., Ashino K., Nagahara T., Gotoh E., Umemura S. et al. (2000) Angiotensin-(1-7) attenuates vasoconstriction evoked by angiotensin ii but not by noradrenaline in man. Hypertension 35, 998–1001 10.1161/01.HYP.35.4.998 10775575

[103] Roks A., van Geel P., Pinto Y., Buikema H., Henning R., de Zeeuw D. et al. (1999) Angiotensin-(1-7) is a modulator of the human renin-angiotensin system. Hypertension 34, 296–301 10.1161/01.HYP.34.2.296 10454457

[104] Freeman E.J., Chisolm G.M., Ferrario C.M. and Tallant E.A. (1996) Angiotensin-(1-7) inhibits vascular smooth muscle cell growth. Hypertension 28, 104–108 10.1161/01.HYP.28.1.104 8675248

[105] Zhang F., Ren X., Zhao M., Zhou B. and Han Y. (2016) Angiotensin-(1-7) abrogates angiotensin II-induced proliferation, migration and inflammation in VSMCs through inactivation of ROS-mediated PI3K/Akt and MAPK/ERK signaling pathways. Sci. Rep. 6, 34621 10.1038/srep34621 27687768 PMC5043354

[106] Akhtar S., Yousif M.H., Dhaunsi G.S., Chandrasekhar B., Al-Farsi O. and Benter I.F. (2012) Angiotensin-(1-7) inhibits epidermal growth factor receptor transactivation via a Mas1receptor-dependent pathway. Br. J. Pharmacol. 165, 1390–1400 10.1111/j.1476-5381.2011.01613.x 21806601 PMC3372724

[107] Skiba D.S., Nosalski R., Mikolajczyk T.P., Siedlinski M., Rios F.J., Montezano A.C. et al. (2017) Anti-atherosclerotic effect of the angiotensin 1-7 mimetic AVE0991 is mediated by inhibition of perivascular and plaque inflammation in early atherosclerosis. Br. J. Pharmacol. 174, 4055–4069 10.1111/bph.13685 27935022 PMC5659999

[108] Yang J., Sun Y., Dong M., Yang X., Meng X., Niu R. et al. (2015) Comparison of angiotensin-(1-7), losartan and their combination on atherosclerotic plaque formation in apolipoprotein E knockout mice. Atherosclerosis 240, 544–549 10.1016/j.atherosclerosis.2015.02.055 25957120

[109] Sui Y.B., Chang J.R., Chen W.J., Zhao L., Zhang B.H., Yu Y.R. et al. (2013) Angiotensin-(1-7) inhibits vascular calcification in rats. Peptides 42, 25–34 10.1016/j.peptides.2012.12.023 23291307

[110] Asahara T., Masuda H., Takahashi T., Kalka C., Pastore C., Silver M. et al. (1999) Bone marrow origin of endothelial progenitor cells responsible for postnatal vasculogenesis in physiological and pathological neovascularization. Circ. Res. 85, 221–228 10.1161/01.RES.85.3.221 10436164

[111] Rodgers K., Xiong S. and diZerega G. (2002) Accelerated recovery from irradiation injury by angiotensin peptides. Cancer Chemother. Pharmacol. 49, 403–411 10.1007/s00280-002-0434-6 11976835

[112] Ellefson D., diZerega G., Espinoza T., Roda N., Maldonado S. and Rodgers K. (2003) Synergistic effects of co-administration of angiotensin 1-7 and Neupogen on hematopoietic recovery in mice. Cancer Chemother. Pharmacol. 53, 15–24 10.1007/s00280-003-0710-0 14569417

[113] Shenoy V., Gjymishka A., Jarajapu Y., Qi Y., Afzal A., Rigatto K. et al. (2013) Diminazene attenuates pulmonary hypertension and improves angiogenic progenitor cell functions in experimental models. Am. J. Respir. Crit. Care Med. 187, 648–657 10.1164/rccm.201205-0880OC 23370913 PMC3733435

[114] Papinska A., Mordwinkin N., Meeks C., Jadhav S. and Rodgers K. (2015) Angiotensin-(1-7) administration benefits cardiac, renal and progenitor cell function in db/db mice. Br. J. Pharmacol. 172, 4443–4453 10.1111/bph.13225 26075703 PMC4562506

[115] Wang Y., Qian C., Roks A., Westermann D., Schumacher S., Escher F. et al. (2010) Circulating rather than cardiac angiotensin-(1-7) stimulates cardioprotection after myocardial infarction. Circ. Heart Fail. 3, 286–293 10.1161/CIRCHEARTFAILURE.109.905968 20103774

[116] Chen J., Xiao X., Chen S., Zhang C., Chen J., Yi D. et al. (2013) Angiotensin-converting enzyme 2 priming enhances the function of endothelial progenitor cells and their therapeutic efficacy. Hypertension 61, 681–689 10.1161/HYPERTENSIONAHA.111.00202 23266545 PMC4011714

[117] Jarajapu Y., Bhatwadekar A., Caballero S., Hazra S., Shenoy V., Medina R. et al. (2012) Activation of the ACE2/Angiotensin-(1-7)/Mas receptor axis enhances the reparative function of dysfunctional diabetic endothelial progenitors. Diabetes 62, 1258–1269 10.2337/db12-0808 23230080 PMC3609564

[118] Singh N., Joshi S., Guo L., Baker M., Li Y., Castellano R. et al. (2015) ACE2/Ang-(1-7)/Mas axis stimulates vascular repair-relevant functions of CD34+cells. Am. J. Physiol. Heart Circ. Physiol. 309, H1697–H1707 26386115 10.1152/ajpheart.00854.2014 PMC4666983

[119] Romero A., San Hipólito-Luengo Á., Villalobos L., Vallejo S., Valencia I., Michalska P. et al. (2019) The angiotensin-(1-7)/Mas receptor axis protects from endothelial cell senescence via klotho and Nrf2 activation. Aging Cell 18, e12913 10.1111/acel.12913 30773786 PMC6516147

[120] Murugan D., Lau Y., Lau W., Mustafa M. and Huang Y. (2015) Angiotensin 1-7 protects against angiotensin II-induced endoplasmic reticulum stress and endothelial dysfunction via Mas1 receptor. Plos One 10, e0145413 10.1371/journal.pone.0145413 26709511 PMC4692500

[121] Xiao X., Zhang C., Ma X., Miao H., Wang J. and Liu L. (2015) Angiotensin-(1-7) counteracts angiotensin II-induced dysfunction in cerebral endothelial cells via modulating Nox2/ROS and PI3K/NO pathways. Exp. Cell Res. 336, 58–63 10.1016/j.yexcr.2015.06.010 26101159 PMC4509813

[122] Pai W., Lo W., Hsu T., Peng C. and Wang H. (2017) Angiotensin-(1-7) inhibits thrombin-induced endothelial phenotypic changes and reactive oxygen species production via NADPH oxidase 5 downregulation. Front. Physiol. 8, 10.3389/fphys.2017.00994 29375391 PMC5770656

[123] Jaiswal N., Diz D.I., Chappell M.C., Khosla M.C. and Ferrario C.M. (1992) Stimulation of endothelial cell prostaglandin production by angiotensin peptides. Characterization of receptors. Hypertension 19, II49–II55 10.1161/01.HYP.19.2_Suppl.II49 1735595

[124] Benter I.F., Ferrario C.M., Morris M. and Diz D.I. (1995) Antihypertensive actions of angiotensin-(1-7) in spontaneously hypertensive rats. Am. J. Physiol. 269, H313–H319 7631863 10.1152/ajpheart.1995.269.1.H313

[125] Clark M., Diz D. and Tallant E. (2001) Angiotensin-(1-7) downregulates the angiotensin II type 1 receptor in vascular smooth muscle cells. Hypertension 37, 1141–1146 10.1161/01.HYP.37.4.1141 11304516

[126] Li N., Cai R., Niu Y., Shen B., Xu J. and Cheng Y. (2012) Inhibition of angiotensin II- induced contraction of human airway smooth muscle cells by angiotensin-(1-7) via downregulation of the RhoA/ROCK2 signaling pathway. Int. J. Mol. Med. 30, 811–818 10.3892/ijmm.2012.1080 22842919

[127] Zhang F., Li S., Song J., Liu J., Cui Y. and Chen H. (2017) Angiotensin-(1-7) regulates angiotensin II-induced matrix metalloproteinase-8 in vascular smooth muscle cells. Atherosclerosis 261, 90–98 10.1016/j.atherosclerosis.2017.02.012 28283184

[128] Bihl J., Zhang C., Zhao Y., Xiao X., Ma X., Chen Y. et al. (2015) Angiotensin-(1-7) counteracts the effects of Ang II on vascular smooth muscle cells, vascular remodelling and hemorrhagic stroke: Role of the NFкB inflammatory pathway. Vascul. Pharmacol. 73, 115–123 10.1016/j.vph.2015.08.007 PMC4617528 26264508

[129] Tan J., Xia J., He H., Chen F., Fan J., Ou S. et al. (2020) Influence of angiotensin- (1-7) on cell activation in rat renal interstitial fibroblasts induced by aldosterone. Xi. Bao. Yu. Fen. Zi. Mian. Yi. Xue. Za. Zhi. 28, 808–810 22863585

[130] Tao X., Fan J., Kao G., Zhang X., Su L., Yin Y. et al. (2014) Angiotensin- (1-7) attenuates angiotensin II-induced signaling associated with activation of a tyrosine phosphatase in Sprague-Dawley rats cardiac fibroblasts. Biol. Cell 106, 182–192 10.1111/boc.201400015 24641355

[131] Chen Y., Fan J., Cao L., Han T., Zeng M., Xu Y. et al. (2019) Unique mechanistic insights into the beneficial effects of angiotensin-(1-7) on the prevention of cardiac fibrosis: A metabolomic analysis of primary cardiac fibroblasts. Exp. Cell Res. 378, 158–170 10.1016/j.yexcr.2019.03.006 30844388

[132] Montezano A.C., Nguyen Dinh Cat A., Rios F.J. and Touyz R.M. (2014) Angiotensin II and vascular injury. Curr. Hypertens. Rep. 16, 431 10.1007/s11906-014-0431-2 24760441

[133] Zhang Z., Chen L., Zhong J., Gao P. and Oudit G. (2014) ACE2/Ang-(1-7) signaling and vascular remodelling. Sci. China Life Sci. 57, 802–808 10.1007/s11427-014-4693-3 25104453

[134] Fraga-Silva R., Costa-Fraga F., Murça T., Moraes P., Martins Lima A., Lautner R. et al. (2013) Angiotensin-Converting Enzyme 2 activation improves endothelial function. Hypertension 61, 1233–1238 10.1161/HYPERTENSIONAHA.111.00627 23608648 PMC3733257

[135] Xu P., Costa-Goncalves A., Todiras M., Rabelo L., Sampaio W., Moura M. et al. (2008) Endothelial dysfunction and elevated blood pressure in mas gene-deleted mice. Hypertension 51, 574–580 10.1161/HYPERTENSIONAHA.107.102764 18180400

[136] Santos R., Castro C., Gava E., Pinheiro S., Almeida A., de Paula R. et al. (2006) Impairment of in vitro and in vivo heart function in angiotensin-(1-7) receptor mas knockout mice. Hypertension 47, 996–1002 10.1161/01.HYP.0000215289.51180.5c 16567589

[137] Simões e Silva A., Silveira K., Ferreira A. and Teixeira M. (2013) ACE2, angiotensin-(1-7) and Mas receptor axis in inflammation and fibrosis. Br. J. Pharmacol. 169, 477–492 10.1111/bph.12159 23488800 PMC3682698

[138] Villalobos L., San Hipólito-Luengo Á., Ramos-González M., Cercas E., Vallejo S., Romero A. et al. (2016) The Angiotensin-(1-7)/Mas axis counteracts angiotensin II-dependent and-independent pro-inflammatory signaling in human vascular smooth muscle cells. Front. Pharmacol. 7, 482 10.3389/fphar.2016.00482 28018220 PMC5156706

[139] Wang L., Hu X., Zhang W. and Tian F. (2012) Angiotensin (1-7) ameliorates angiotensin II-induced inflammation by inhibiting LOX-1 expression. Inflam. Res. 62, 219–228 10.1007/s00011-012-0571-2 23233095

[140] Zhang Y., Zhang Y., Dong X., Hao Q., Zhou X., Yu Q. et al. (2015) ACE2 and Ang-(1-7) protect endothelial cell function and prevent early atherosclerosis by inhibiting inflammatory response. Inflamm. Res. 64, 253–260 10.1007/s00011-015-0805-1 25721616

[141] Nguyen Dinh Cat A., Montezano A., Burger D. and Touyz R. (2013) Angiotensin II, NADPH oxidase, and redox signaling in the vasculature. Antioxid. Redox Signal. 19, 1110–1120 10.1089/ars.2012.4641 22530599 PMC3771549

[142] Zhang Y., Liu J., Luo J., Tian X., Cheang W., Xu J. et al. (2015) Upregulation of Angiotensin (1-7)-mediated signaling preserves endothelial function through reducing oxidative stress in diabetes. Antioxid. Redox Signal. 23, 880–892 10.1089/ars.2014.6070 25867182 PMC4617412

[143] Lin L., Liu X., Xu J., Weng L., Ren J., Ge J. et al. (2015) Mas receptor mediates cardioprotection of angiotensin-(1-7) against Angiotensin II-induced cardiomyocyte autophagy and cardiac remodelling through inhibition of oxidative stress. J. Cell. Mol Med. 20, 48–57 10.1111/jcmm.12687 26515045 PMC4717848

[144] Wang J., Chen S. and Bihl J. (2020) Exosome-mediated transfer of ACE2 (angiotensin-converting enzyme 2) from endothelial progenitor cells promotes survival and function of endothelial cell. Oxid. Med. Cell. Longev. 2020, 1–11 10.1155/2020/4213541 PMC6995312 32051731

[145] Arroja M., Reid E., Roy L., Vallatos A., Holmes W., Nicklin S. et al. (2019) Assessing the effects of Ang-(1-7) therapy following transient middle cerebral artery occlusion. Sci. Rep. 9, 3154 10.1038/s41598-019-39102-8 30816157 PMC6395816

[146] Benter I., Yousif M., Dhaunsi G., Kaur J., Chappell M. and Diz D (2007) Angiotensin-(1-7) prevents activation of NADPH oxidase and renal vascular dysfunction in diabetic hypertensive rats. Am. J. Nephrol. 28, 25–33 10.1159/000108758 17890855

[147] Ma Y., Huang H., Jiang J., Wu L., Lin C., Tang A. et al. (2016) AVE 0991 attenuates cardiac hypertrophy through reducing oxidative stress. Biochem. Biophysic. Res. Commun. 474, 621–625 10.1016/j.bbrc.2015.09.050 26403967

[148] Rathinasabapathy A., Bryant A., Suzuki T., Moore C., Shay S., Gladson S. et al. (2018) rhACE2 therapy modifies bleomycin-induced pulmonary hypertension via rescue of vascular remodeling. Front. Physiol. 9, 271 10.3389/fphys.2018.00271 29731719 PMC5922219

[149] Guo L., Yin A., Zhang Q., Zhong T., O'Rourke S. and Sun C. (2017) Angiotensin-(1-7) attenuates angiotensin II-induced cardiac hypertrophy via a Sirt3-dependent mechanism. Am. J. Physiol. Heart Circ. Physiol. 312, H980–H991 10.1152/ajpheart.00768.2016 28411231

[150] Fang Y., Gao F. and Liu Z. (2019) Angiotensin-converting enzyme 2 attenuates inflammatory response and oxidative stress in hyperoxic lung injury by regulating NF-κB and Nrf2 pathways. QJM-Int. J. Med. 112, 914–924 10.1093/qjmed/hcz206 31393582

[151] Gwathmey T., Pendergrass K., Reid S., Rose J., Diz D. and Chappell M. (2010) Angiotensin-(1-7)-angiotensin-converting enzyme 2 attenuates reactive oxygen species formation to angiotensin II within the cell nucleus. Hypertension 55, 166–171 10.1161/HYPERTENSIONAHA.109.141622 19948986 PMC2821807

[152] Matavelli L., Huang J. and Siragy H. (2011) Angiotensin AT2 receptor stimulation inhibits early renal inflammation in renovascular hypertension. Hypertension 57, 308–313 10.1161/HYPERTENSIONAHA.110.164202 21189405 PMC3060557

[153] de Castro C., Souza dos Santos R., Ferreira A., Bader M., Alenina N. and Pinto de Almeida A. (2005) Evidence for a functional interaction of the angiotensin-(1-7) receptor mas with AT 1 and AT 2 receptors in the mouse heart. Hypertension 46, 937–942 10.1161/01.HYP.0000175813.04375.8a 16157793

[154] Patel S., Ali Q., Samuel P., Steckelings U. and Hussain T. (2017) Angiotensin II type 2 receptor and receptor mas are colocalized and functionally interdependent in obese zucker rat kidney. Hypertension 70, 831–838 10.1161/HYPERTENSIONAHA.117.09679 28827476 PMC5599348

[155] Leonhardt J., Villela D., Teichmann A., Münter L., Mayer M., Mardahl M. et al. (2017) Evidence for heterodimerization and functional interaction of the angiotensin type 2 receptor and the receptor mas. Hypertension 69, 1128–1135 10.1161/HYPERTENSIONAHA.116.08814 28461604

[156] Villela D., Leonhardt J., Patel N., Joseph J., Kirsch S., Hallberg A. et al. (2014) Angiotensin type 2 receptor (AT2R) and receptor Mas: a complex liaison. Clin. Sci. (Lond.) 128, 227–234 10.1042/CS20130515 25328009

[157] Roks A., Nijholt J., van Buiten A., van Gilst W., de Zeeuw D. and Henning R. (2004) Low sodium diet inhibits the local counter-regulator effect of angiotensin-(1-7) on angiotensin II. J. Hypertens. 22, 2355–2361 10.1097/00004872-200412000-00018 15614030

[158] Durand M., Raffai G., Weinberg B. and Lombard J. (2010) Angiotensin-(1-7) and low-dose angiotensin II infusion reverse salt-induced endothelial dysfunction via different mechanisms in rat middle cerebral arteries. Am. J. Physiol. Heart Circ. Physiol. 299, H1024–H1033 10.1152/ajpheart.00328.2010 20656887 PMC2957344

[159] Liu Y., Li B., Wang X., Li G., Shang R., Yang J. et al. (2015) Angiotensin-(1-7) suppresses hepatocellular carcinoma growth and angiogenesis via complex interactions of angiotensin II type 1 receptor, angiotensin II type 2 receptor and mas receptor. Mol. Med. 21, 626–636 10.2119/molmed.2015.00022 26225830 PMC4656199

[160] Tesanovic S., Vinh A., Gaspari T., Casley D. and Widdop R. (2010) Vasoprotective and atheroprotective effects of angiotensin (1-7) in apolipoprotein e-deficient mice. Arterioscler. Thromb. Vasc. Biol. 30, 1606–1613 10.1161/ATVBAHA.110.204453 20448208

[161] Mecca A., Regenhardt R., O'Connor T., Joseph J., Raizada M., Katovich M. et al. (2011) Cerebroprotection by angiotensin-(1-7) in endothelin-1-induced ischaemic stroke. Exp. Physiol. 96, 1084–1096 10.1113/expphysiol.2011.058578 21685445 PMC3210510

[162] Jiang T., Gao L., Zhu X., Yu J., Shi J., Tan M. et al. (2013) Angiotensin-(1-7) inhibits autophagy in the brain of spontaneously hypertensive rats. Pharmacol. Res. 71, 61–68 10.1016/j.phrs.2013.03.001 23499735

[163] Brosnihan K., Li P. and Ferrario C. (1996) Angiotensin-(1-7) dilates canine coronary arteries through kinins and nitric oxide. Hypertension 27, 523–528 10.1161/01.HYP.27.3.523 8613197

[164] Silva D., Vianna H., Cortes S., Campagnole-Santos M., Santos R. and Lemos V. (2007) Evidence for a new angiotensin-(1-7) receptor subtype in the aorta of Sprague-Dawley rats. Peptides 28, 702–707 10.1016/j.peptides.2006.10.007 17129638

[165] Wagenaar G., Laghmani E., Fidder M., Sengers R., de Visser Y., de Vries L. et al. (2013) Agonists of MAS oncogene and angiotensin II type 2 receptors attenuate cardiopulmonary disease in rats with neonatal hyperoxia-induced lung injury. Am. J. Physiol. Lung Cell Mol. Physiol. 305, L341–L351 10.1152/ajplung.00360.2012 23812633 PMC3763032

[166] Bruce E., Shenoy V., Rathinasabapathy A., Espejo A., Horowitz A., Oswalt A. et al. (2015) Selective activation of angiotensin AT2 receptors attenuates progression of pulmonary hypertension and inhibits cardiopulmonary fibrosis. Br. J. Pharmacol. 172, 2219–2231 10.1111/bph.13044 25522140 PMC4403089

[167] Costa M., Lopez Verrilli M., Gomez K., Nakagawa P., Peña C., Arranz C. et al. (2010) Angiotensin-(1-7) upregulates cardiac nitric oxide synthase in spontaneously hypertensive rats. Am. J. Physiol. Heart Circ. Physiol. 299, H1205–H1211 10.1152/ajpheart.00850.2009 20675563

[168] Walters P., Gaspari T. and Widdop R. (2005) Angiotensin-(1-7) acts as a vasodepressor agent via angiotensin ii type 2 receptors in conscious rats. Hypertension 45, 960–966 10.1161/01.HYP.0000160325.59323.b8 15767466

[169] Goru S., Kadakol A., Malek V., Pandey A., Sharma N. and Gaikwad A. (2017) Diminazene aceturate prevents nephropathy by increasing glomerular ACE2 and AT2receptor expression in a rat model of type1 diabetes. Br. J. Pharmacol. 174, 3118–3130 10.1111/bph.13946 28688122 PMC5573423

[170] Yamazato Y., Ferreira A., Hong K., Sriramula S., Francis J., Yamazato M. et al. (2009) Prevention of pulmonary hypertension by angiotensin-converting enzyme 2 gene transfer. Hypertension 54, 365–371 10.1161/HYPERTENSIONAHA.108.125468 19564552 PMC2732127

[171] Lu R., Zhao X., Li J., Niu P., Yang B., Wu H. et al. (2020) Genomic characterisation and epidemiology of 2019 novel coronavirus: implications for virus origins and receptor binding. Lancet 395, 565–574 10.1016/S0140-6736(20)30251-8 32007145 PMC7159086

[172] Huang Y., Yang C., Xu X., Xu W. and Liu S. (2020) Structural and functional properties of SARS-CoV-2 spike protein: potential antivirus drug development for COVID-19. Acta Pharmacol. Sin. 41, 1141–1149 10.1038/s41401-020-0485-4 32747721 PMC7396720

[173] Heurich A., Hofmann-Winkler H., Gierer S., Liepold T., Jahn O. and Pohlmann S. (2013) TMPRSS2 and ADAM17 cleave ACE2 differentially and only proteolysis by TMPRSS2 augments entry driven by the severe acute respiratory syndrome coronavirus spike protein. J. Virol. 88, 1293–1307 10.1128/JVI.02202-13 24227843 PMC3911672

[174] Bestle D., Heindl M., Limburg H., Van Lam van T., Pilgram O., Moulton H. et al. (2020) TMPRSS2 and furin are both essential for proteolytic activation of SARS-CoV-2 in human airway cells. Life Sci. Alliance. 3, e202000786 10.26508/lsa.202000786 32703818 PMC7383062

[175] Baruah C., Devi P. and Sharma D (2020) Sequence analysis and structure prediction of SARS-CoV-2 accessory proteins 9b and ORF14: Evolutionary analysis indicates close relatedness to bat coronavirus. BioMed Res. Int. 2020, 1–13 10.1155/2020/7234961 PMC7576348 33102591

[176] Wang K., Chen W., Zhang Z., Deng Y., Lian J., Du P. et al. (2020) CD147-spike protein is a novel route for SARS-CoV-2 infection to host cells. Signal Transduct Target Ther 5, 1–10 10.1038/s41392-020-00426-x 33277466 PMC7714896

[177] Chen Z., Mi L., Xu J., Yu J., Wang X., Jiang J. et al. (2005) Function of HAb18G/CD147 in invasion of host cells by severe acute respiratory syndrome coronavirus. J. Infect. Dis. 191, 755–760 10.1086/427811 15688292 PMC7110046

[178] Ahmetaj-Shala B., Vaja R., Atanur S., George P., Kirkby N. and Mitchell J. (2020) Cardiorenal tissues express SARS-CoV-2 entry genes and basigin (BSG/CD147) increases with age in endothelial cells. J. Am. Coll. Cardiol. Basic Trans. Science. 5, 1111–1123 10.1016/j.jacbts.2020.09.010 PMC7546186 33073064

[179] Soldatov V., Kubekina M., Silaeva Y., Bruter A. and Deykin A. (2020) On the way from SARS-CoV-sensitive mice to murine COVID-19 model. Res. Results Pharmacol. 6, 1–7 10.3897/rrpharmacology.6.53633

[180] Winkler E., Bailey A., Kafai N., Nair S., McCune B., Yu J. et al. (2020) SARS-CoV-2 infection of human ACE2-transgenic mice causes severe lung inflammation and impaired function. Nat. Immunol. 21, 1327–1335 10.1038/s41590-020-0778-2 32839612 PMC7578095

[181] Nishiga M., Wang D.W., Han Y., Lewis D.B. and Wu J.C. (2020) COVID-19 and cardiovascular disease: from basic mechanisms to clinical perspectives. Nat. Rev. Cardiol. 17, 543–558 10.1038/s41569-020-0413-9 32690910 PMC7370876

[182] Guzik T.J., Mohiddin S.A., Dimarco A., Patel V., Savvatis K., Marelli-Berg F. et al. (2020) COVID-19 and the cardiovascular system: implications for risk assessment, diagnosis, and treatment options. Cardiovasc. Res. 116, 1666–1687 10.1093/cvr/cvaa106 32352535 PMC7197627

[183] Zhang P., Zhu L., Cai J., Lei F., Qin J., Xie J. et al. (2020) Association of inpatient use of angiotensin-converting enzyme inhibitors and angiotensin ii receptor blockers with mortality among patients with hypertension hospitalized with COVID-19. Circ. Res. 126, 1671–1681 10.1161/CIRCRESAHA.120.317134 32302265 PMC7265882

[184] Sardu C., Gambardella J., Morelli M., Wang X., Marfella R. and Santulli G. (2020) Hypertension, thrombosis, kidney Failure, and diabetes: Is COVID-19 an endothelial disease? A comprehensive evaluation of clinical and basic evidence J. Clin. Med. 9, 1417 10.3390/jcm9051417 PMC7290769 32403217

[185] Liu P.P., Blet A., Smyth D. and Li H. (2020) The science underlying COVID-19: Implications for the cardiovascular system. Circulation 142, 68–78 10.1161/CIRCULATIONAHA.120.047549 32293910

[186] Zheng Y.Y., Ma Y.T., Zhang J.Y. and Xie X. (2020) COVID-19 and the cardiovascular system. Nat. Rev. Cardiol. 17, 259–260 10.1038/s41569-020-0360-5 32139904 PMC7095524

[187] Teuwen L., Geldhof V., Pasut A. and Carmeliet P. (2020) COVID-19: the vasculature unleashed. Nat. Rev. Immunol. 20, 389–391 10.1038/s41577-020-0343-0 32439870 PMC7240244

[188] Varga Z., Flammer A., Steiger P., Haberecker M., Andermatt R., Zinkernagel A. et al. (2020) Endothelial cell infection and endotheliitis in COVID-19. Lancet 395, 1417–1418 10.1016/S0140-6736(20)30937-5 32325026 PMC7172722

[189] Oudit G.Y., Kassiri Z., Jiang C., Liu P.P., Poutanen S.M., Penninger J.M. et al. (2009) SARS-coronavirus modulation of myocardial ACE2 expression and inflammation in patients with SARS. Eur. J. Clin. Invest. 39, 618–625 10.1111/j.1365-2362.2009.02153.x 19453650 PMC7163766

[190] Scialo F., Daniele A., Amato F., Pastore L., Matera M., Cazzola M. et al. (2020) ACE2: The major cell entry receptor for SARS-CoV-2. Lung 198, 867–877 10.1007/s00408-020-00408-4 33170317 PMC7653219

[191] Kaur U., Acharya K., Mondal R., Singh A., Saso L., Chakrabarti S. et al. (2020) Should ACE2 be given a chance in COVID-19 therapeutics: A semi-systematic review of strategies enhancing ACE2. Eur. J. Pharmacol. 887, 173545 32926917 10.1016/j.ejphar.2020.173545 PMC7485553

[192] Zhang H., Penninger J., Li Y., Zhong N. and Slutsky A. (2020) Angiotensin-converting enzyme 2 (ACE2) as a SARS-CoV-2 receptor: molecular mechanisms and potential therapeutic target. Intensive Care Med. 46, 586–590 32125455 10.1007/s00134-020-05985-9 PMC7079879

[193] Groß S., Jahn C., Cushman S., Ba¨ r C. and Thum T. (2020) SARS-CoV-2 receptor ACE2-dependent implications on the cardiovascular system: From basic science to clinical implications. J. Mol. Cell. Cardiol. 144, 47–53 32360703 10.1016/j.yjmcc.2020.04.031 PMC7191280

[194] Monteil V., Kwon H., Prado P., Hagelkrüys A., Wimmer R., Stahl M. et al. (2020) Inhibition of SARS-CoV-2 infections in engineered human tissues using clinical-grade soluble human ACE2. Cell 181, 905.e7–913.e7 32333836 10.1016/j.cell.2020.04.004 PMC7181998

[195] Batlle D., Wysocki J. and Satchell K. (2020) Soluble angiotensin-converting enzyme 2: a potential approach for coronavirus infection therapy? Clin. Sci. (Lond.) 134, 543–545 32167153 10.1042/CS20200163

